# Cells, Materials, and Fabrication Processes for Cardiac Tissue Engineering

**DOI:** 10.3389/fbioe.2020.00955

**Published:** 2020-08-11

**Authors:** Pilar Montero, María Flandes-Iparraguirre, Saioa Musquiz, María Pérez Araluce, Daniel Plano, Carmen Sanmartín, Gorka Orive, Juan José Gavira, Felipe Prosper, Manuel M. Mazo

**Affiliations:** ^1^Regenerative Medicine Program, Cima Universidad de Navarra, Foundation for Applied Medical Research, Pamplona, Spain; ^2^NanoBioCel Group, Laboratory of Pharmaceutics, School of Pharmacy, University of the Basque Country – UPV/EHU, Vitoria-Gasteiz, Spain; ^3^Department of Pharmaceutical Technology and Chemistry, University of Navarra, Pamplona, Spain; ^4^IdiSNA, Navarra Institute for Health Research, Pamplona, Spain; ^5^University Institute for Regenerative Medicine and Oral Implantology – UIRMI (UPV/EHU – Fundación Eduardo Anitua), Vitoria-Gasteiz, Spain; ^6^Singapore Eye Research Institute, Singapore, Singapore; ^7^Cardiology Department, Clínica Universidad de Navarra, Pamplona, Spain; ^8^Hematology and Cell Therapy Area, Clínica Universidad de Navarra, Pamplona, Spain

**Keywords:** cardiac tissue engineering, human pluripotent stem cells, material properties, cell differentiation, fabrication strategies

## Abstract

Cardiovascular disease is the number one killer worldwide, with myocardial infarction (MI) responsible for approximately 1 in 6 deaths. The lack of endogenous regenerative capacity, added to the deleterious remodelling programme set into motion by myocardial necrosis, turns MI into a progressively debilitating disease, which current pharmacological therapy cannot halt. The advent of Regenerative Therapies over 2 decades ago kick-started a whole new scientific field whose aim was to prevent or even reverse the pathological processes of MI. As a highly dynamic organ, the heart displays a tight association between 3D structure and function, with the non-cellular components, mainly the cardiac extracellular matrix (ECM), playing both fundamental active and passive roles. Tissue engineering aims to reproduce this tissue architecture and function in order to fabricate replicas able to mimic or even substitute damaged organs. Recent advances in cell reprogramming and refinement of methods for additive manufacturing have played a critical role in the development of clinically relevant engineered cardiovascular tissues. This review focuses on the generation of human cardiac tissues for therapy, paying special attention to human pluripotent stem cells and their derivatives. We provide a perspective on progress in regenerative medicine from the early stages of cell therapy to the present day, as well as an overview of cellular processes, materials and fabrication strategies currently under investigation. Finally, we summarise current clinical applications and reflect on the most urgent needs and gaps to be filled for efficient translation to the clinical arena.

## A Perspective on Cardiac Disease and Regenerative Medicine

Organ transplantation is one of the greatest medical achievements of the 20th century. However, its applicability is hampered by donor shortage, life-long immunosuppression and its success rates are linked to the experience of the surgical team. It requires a well-coordinated national effort, which is sometimes hindered by ethical issues (Prabhu, 2019). The search for novel ways to approach organ repair inspired the field of regenerative medicine, with Stem Cell Therapy as one of the most representative examples. Since this began, stem cells have been discovered even in low turnover adult tissues, such as the central nervous system (Doetsch et al., 1999), the lung, (Rock et al., 2009; Barkauskas et al., 2013) or the heart, (Beltrami et al., 2003) and have been widely assayed in animal models of disease, quickly reaching clinical trials. This swift progression in general met with rapid failure, but on the bright side, it also enabled specialists to gain immense insights into their mechanisms and ways of action.

Nothing exemplifies this journey better than the cardiac field. Cardiovascular diseases are well recognised as the leading cause of death worldwide, accounting for almost 1 in 2 deaths in Europe and causing 3.9 million deaths per year (Townsend et al., 2016). Ischemic heart disease (IHD) is one shade on this spectrum. It is generally caused by the clotting of a coronary vessel, which in turn leads to the death of a portion of the myocardium and the subsequent functional impairment of the organ. Being mostly non-regenerative, the heart is chronically impaired (Eschenhagen et al., 2017). A real epidemic, IHD is the leading single cause of death globally, responsible for over 15 million deaths in 2016, ranking first in high- and lower-middle-income countries, and third in low-income countries (World Health Organization [WHO], 2018). As per 2015, over 22 million EU citizens were living with the disease, with approximately 3 million new cases yearly. IHD imposes an enormous burden on society as affected patients must be cared for by health systems, requiring lifelong highly specialised medical attention and multimedication. It also jeopardises the structure of the workforce and puts significant pressure on families. In terms of economic cost, the total burden of IHD for EU economies is estimated at €59 billion/year. Of these, €19 billion is directly related to healthcare costs, while €20 billion is linked to productivity losses and the remaining €20 billion to the cost of indirect care. Estimations sketch out a grim future. In the United States, heart attacks are projected to contribute more than $818 billion to annual healthcare costs and lost productivity by 2030, (Nowbar et al., 2019) while in the South Asia region, direct medical costs for CVD are estimated to reach US $16.6 billion in 2021 (Walker et al., 2018). Organ transplant cannot match this overwhelming demand and become a widespread therapeutic option (Stehlik et al., 2011).

It was hoped that cell therapy would provide new means to regenerate the scarred myocardium. Remarkable discoveries encouraged rapid progression towards clinical trials, with the first one launched in record time (Behfar et al., 2014). After the BOOST and REPAIR-AMI trials reported significant benefits in cardiac function, (Wollert et al., 2004; Schächinger et al., 2006) hundreds of patients were recruited often in individual efforts mostly based on local experience. This led to the first voicing of concerns. The primary initial objective, improvement of cardiac function, failed to be met in many cases. Although statistically significant benefits in cardiac function were sometimes found, their magnitude was not as great as had been expected (reviewed in Gyöngyösi et al., 2016; Menasché, 2018). This reversal of fortunes coincided also with a remarkable twist in our understanding of what basic science and animal models conveyed: no true regeneration of the myocardium was achieved by adult stem cells. The underlying effects were mostly due to the paracrine secretion of beneficial molecules (Hodgkinson et al., 2016). Although this general perspective is valid for most adult stem cells, use of their embryonic counterpart, although boosted by their capacity to give rise to new tissue once transplanted, was still marred by some common issues such as lack of proper engraftment, and crucially by safety concerns as teratoma formation and need for immune suppression, as well as ethical issues.

Did stem cell regenerative medicine approaches fail? They obviously did not achieve the initial aims, but looking back, those can no doubt be branded as overambitious (Pagano et al., 2019). It did succeed in gathering a whole new compendium of knowledge, which has led to renewed and better efforts in the regenerative direction and, importantly, has built a worldwide network of excellence encompassing not only different nationalities, but also very diverse scientific disciplines. One of the greatest and perhaps now evident realisations in the field is the notion that cells are not alone in a tissue, and that the extracellular matrix (ECM) has a predominant role, not only as a passive architectural element, but crucially as a signal transducer and determinant of functionality (Majkut et al., 2013; Crowder et al., 2016; Kumar et al., 2017). Specifically in the myocardium, the ECM is highly dynamic, changing during development and disease. This latter change is bidirectional, as disease induces pathological ECM deposition but an abnormal matrix is able to produce malfunction (Frangogiannis, 2019). In consequence, it is now recognised that a cell-based cardiac regeneration without an adequate ECM is not viable. Generating new myocardium thus requires the participation of the most promising cells, with a surrounding matrix able to replicate the conditions of the native tissue and the proper 3D architecture. This is precisely one of the main directions of cardiac Tissue Engineering.

Cardiac Tissue Engineering (cTE) is a highly interdisciplinary scientific discipline, aiming at reproducing as accurately as possible the function and biology of cardiac muscle, during development or maturity, health or disease. Although its first objective was focused on meeting the needs of cardiac regenerative Medicine, as knowledge and experience on how the ECM influences cardiac cell biology increased and the fabrication capacities widened, its scope has greatly expanded into areas such as disease modelling, drug testing and personalised medicine amongst others (Feric et al., 2019; Noor et al., 2019; Mastikhina et al., 2020). This review aims at presenting the reader with an overview of the specific characteristics of the myocardium that determine the needs regenerative cTE has to meet, as well as providing a non-exhaustive revision of what the field has delivered to attain this end, with a focus on human myocardium and human pluripotent stem cells.

## The Heart

The mammalian heart is an incredible organ. Its main role is to provide a continuous unidirectional supply of blood to the organism. This comes at a stringent metabolic cost, consuming the equivalent of 6 kg of ATP per day, with a complete renewal of its ATP pool every 10 seconds. Most of this energy is obtained through the oxidation of fatty acids in adulthood, though cardiac metabolism is dependent on glucose during embryonic stages, being able to employ lactate as a metabolic substrate (Neejy, 1974). Correct function is achieved through a specialised organ architecture, dividing the heart into 4 chambers: atria, which are smaller in size and muscular mass, receive blood and push it out into the ventricles, which in turn pump either towards the lungs or the body. In consequence, the left ventricle is larger and has a thicker muscular wall than its right counterpart. Chambers, inlets and outlets are separated by valves impeding back flow. The heart is the first organ to function, around day 8 in mice and the 4^th^ gestational week in humans (Brand, 2003). It pumps continuously throughout life, efficiently ejecting blood through an exquisite 3D structure, (Buckberg, 2002) established by a complex set of embryonic movements, cellular growth and incorporation (Günthel et al., 2018). Disruption of this structure is seen in disease and can be in itself the cause of organ malfunction: cardiac congenital defects and malformation are a main cause of perinatal death, (Bressan et al., 2013) but also give rise to many cardiomyopathies (McKenna et al., 2017).

### Cardiac Embryonic Development: A Brief Overview

The formation of the mammalian four-chambered heart encompasses a series of tightly coordinated morphological, cellular and molecular events (reviewed in Vincent and Buckingham, 2010; Meilhac et al., 2014; Ruiz-Villalba et al., 2016). Different pools of cardiac and extracardiac progenitors are involved, including the mesoderm-derived First, Second and Third Heart Fields (FHF, SHF and THF respectively) and the Cardiac Neural Crest Cells (CNCCs). Cells of the FHF contribute primarily to the left ventricle (LV) but there is also a small contribution to the atria; SHF will form the right ventricle (RV), outflow tract (OFT), atria and part of inflow tract (IFT); (Buckingham et al., 2005). THF cells contribute to the sinus node, some regions in the caval myocardium, and the Pro-Epicardial Organ (PEO) (Mommersteeg et al., 2010; Bressan et al., 2013). CNCCs arise from the dorsal neural tube, contribute to the parasympathetic innervation of the heart, valves and play a pivotal role in OFT patterning and optimal septation (Keyte et al., 2014).

Early precardiac progenitors from the lateral mesoderm have been mapped into the mid-anterior region of the primitive streak, characterised by the presence of both anterior Nodal/Activin and posterior bone morphogenetic protein (BMP) signalling at low levels, (Zhang et al., 2008; Vallier et al., 2009; Yamauchi et al., 2010; Yu et al., 2011) promoting the emergence of cardiogenic mesodermal MIXL1 + KDR + cells. As a result of these signalling gradients set in gastrulation, multipotent cardiovascular progenitor (M) expressing the cardiac master regulator MESP1 move in an anterior-lateral direction, forming a horseshoe-like region termed the cardiac crescent or FHF (Bondue et al., 2008; Chan S. S. K. et al., 2013). At a molecular level, MESP1 induces the expression of the minimal core of the essential cardiogenic transcription factors including ISL1, TBX5, NKX2.5, and GATA4, in combination with the chromatin remodeller SMARCD3 (BAF60C), which further drive cardiomyogenesis (Vincent and Buckingham, 2010; Meilhac et al., 2014; Meilhac and Buckingham, 2018). The cardiac crescent fuses at the midline, forming the linear heart tube, which consists of an interior layer of endocardial cells and an exterior layer of myocardial cells separated by an acellular, ECM-rich space, the cardiac jelly. Located central and dorsal to the FHF, SHF cells remain in contact with the pharyngeal endoderm and in a proliferative state as undifferentiated ISL1 + MEF2C + cells. As development proceeds, SHF cells are added to the poles of the heart tube, with the tube looping to position the different regions into place. Chambers balloon out as a result of the differential proliferation rates of CMs (Jong et al., 1997; Christoffels et al., 2004). As already mentioned, THF cells (TBX18 + NKX2.5-) contribute to the sinus node, caval myocardial cells and the PEO. PEO-cells give rise to the epicardium, and some cells of this layer undergo epithelial-to-mesenchymal transition to form epicardial derived cells (EPDC), which will differentiate into vascular cells (including the coronaries) as well as interstitial fibroblasts and valvular cells, being essential for compaction (Pérez-Pomares et al., 2002; Weeke-Klimp et al., 2010; Katz et al., 2012). Lastly, CNCCs originate by delamination from the neuroectoderm, (Hildreth et al., 2008) initially contributing to smooth muscle cells and CMs, (Mjaatvedt et al., 2001) and making a significant contribution to the innervation of the organ and to the OFT (Hildreth et al., 2008; Sizarov et al., 2012). We refer the reader to Table 1 for a full description of the mentioned gene abbreviations.

**TABLE 1:** Full description of genes names.

Abbreviation	Description
MIXL1	Mix Paired-Like Homeobox
KDR	Kinase Insert Domain Receptor
MESP1	Mesoderm Posterior BHLH Transcription Factor 1
ISL1	Islet-1 LIM Homeobox
TBX5	T-Box Transcription Factor 5
NKX2.5	NK2 homeobox 5
GATA4	GATA Binding Protein 4
SMARCD3	SWI/SNF Related, Matrix Associated, Actin Dependent Regulator Of Chromatin, Subfamily D, Member 3
BX18	T-Box Transcription Factor 18

### Post-birth Cardiac Development: Foetal CMs vs. Adult CMs

Aside from the formation of the mammalian heart, CMs continue to develop postnatally (Guo and Pu, 2020). Embryonic CMs can beat spontaneously, express sarcomeric proteins and ion channels, and exhibit action potentials and calcium transients which are significantly distinctive from their adult counterpart (Vincent and Buckingham, 2010; Meilhac et al., 2014). Human and rodent embryonic CMs are around 30-40 fold less in size and feature an irregular shape, in comparison with adult CMs (Yang et al., 2014). These are characterised by an ultrastructural organisation with a large mitochondrial volume and specific mitochondria positioning between myofibrils. Sarcomeres in postnatal CMs are long and well-aligned, in contrast to shorter and disarrayed ones found in foetal CM. At a metabolic level, embryonic CMs rely on glycolysis, whereas adult myocytes preferentially consume fatty acids, a much more efficient energy source. Myofibrillar protein isoform undergoes switching, being myosin heavy chain 7 (MYH7), myosin light chain 2 ventricular isoform (MLC2v), cardiac troponin I3 (TNNI3) and a shorter and stiffer Titin isoform, preferentially expressed in adult CMs, in contrast to myosin heavy chain 6 (MYH6), myosin light chain 2 atrial isoform (MLC2a), and slow skeletal-type troponin I1 (TNNI1) on foetal CMs (Bedada et al., 2014). All these differences directly correlate with contractile capacity, with adult CMs able to generate more force than embryonic ones (Vincent and Buckingham, 2010; Meilhac et al., 2014; Tan and Ye, 2018). For example, strips of adult rat myocardium have been reported to produce a peak twitch tension of 56.4 ± 44 mN/mm2, (Hasenfuss et al., 1991) whereas collagen constructs with neonatal rat CMs generated 0.4-0.8 mN/mm2 (Zimmermann et al., 2002). The same difference in magnitude is believed to exist for human cells, as comparisons with primary foetal human CMs are rare (Yang et al., 2014).

The cardiac action potential and associated channels and currents also distinguishes adult and foetal CMs. In immature CMs, the expression of channels involved in repolarisation, including potassium transient outward channels, L-type calcium currents and the rectifying K + current (encoded mainly by KCNJ2), is lower than in adult cells resulting in a less negative resting membrane potential (−50mv ∼−60 mv in embryonic CMs) compared to normal (–85mv ∼ –90 mv in adult CMs) (Zhang et al., 2009). Also, the pacemaker current I_f_ is present in embryonic CMs but does not occur in adult myocytes (Sartiani et al., 2007). The distribution of the gap junction protein connexin 43 (Cx43) also plays an important role in regulating electrical activity. While Cx43 concentrates at the intercalated disc of adult CMs, it is circumferentially distributed in immature CMs, which is not optimal for longitudinal electrical propagation (Vreeker et al., 2014; Jiang et al., 2018). Adult CMs have a well-developed sarcoplasmic reticulum (SR) with a high level of SR-specific proteins like ryanodine receptor 2 (RYR2) and sarcoplasmic/endoplasmic reticulum Ca2 + ATPase 2a (SERCA2), (Ivashchenko et al., 2013) which, coupled with the presence of transverse tubules (t-tubules), leads to a highly coordinated Ca-induced-Ca-release and hence faster Ca transient kinetics and amplitude when compared to foetal CMs (Louch et al., 2015). Finally, where embryonic CMs are diploid, adult CMs present different degrees of polyploidy, achieved through DNA-synthesis without karyokinesis (Adler and Costabel, 1975; Herget et al., 1997). Understanding how an embryonic CM evolves into a mature cell is already proving fundamental in human cTE. As the bioartificial tissues developed so far resemble more their foetal counterpart, this insight is being incorporated into the effort of driving engineered tissues towards an adult-like functionality (Karbassi et al., 2020).

### Heart Characteristics: What We Aim to Engineer

**FIGURE 1:**
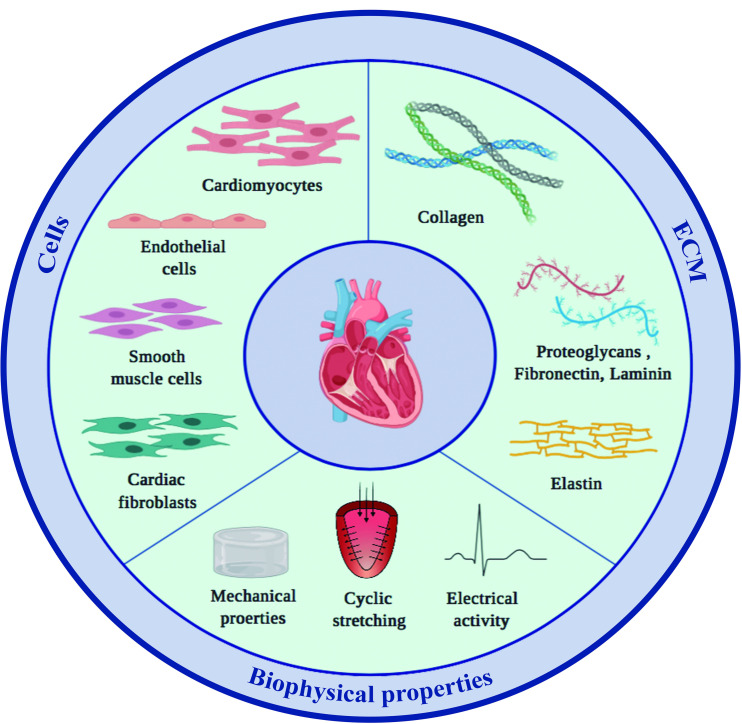
Main components of the mammalian heart. The two main constituents of the myocardium, cardiac cells and the surrounding ECM, both contribute to and modulate the specific material properties of the tissue.

Generating human myocardial surrogates in the laboratory requires knowing what the natural composition and properties of the organ are. The following paragraphs provide an overview of what nature has achieved, specifically, what the main cellular and extracellular components of the heart are, how they are arranged in space, and importantly, what this means regarding the resulting material properties (Figure 1).

#### Cellular Composition

As already explained, most cells forming the structure of the heart are of mesodermal origin: CMs, vascular (endothelial and smooth muscle) cells and fibroblasts. Others reside in the tissue but are formed elsewhere, as immune cells, which play a significant role in organ surveillance and disease. Deciphering the cellular composition of the heart has been a very controversial subject, be it in rodents or humans (Zhou and Pu, 2016). Histology can determine that CMs are the largest fraction by volume. However, numbers vary, with reports of murine myocytes being the largest population by number [56/27/7 for CMs/fibroblasts/endothelial cells respectively (Banerjee et al., 2007)] and others attributing greater numbers to endothelial cells [43.6% vs 31% of CMs (Pinto et al., 2016)]. Human proportions are similarly contradictory, with some publications showing non-CM/endothelial cells are the most abundant (Bergmann et al., 2015) and others endothelial cells (Anversa et al., 1978). Furthermore, several studies have reported varying cell proportions throughout the anatomical regions of the organ (Sussman et al., 2002; Gaudesius et al., 2003; Camelliti et al., 2004, 2005; Kohl, 2004; Baudino et al., 2006). Things become more complicated if we take into account the age of the individual, as some claim the final number of myocytes is reached by one month, remaining constant over the lifetime of the individual (Bergmann et al., 2015) whereas others have reported a 3.4-fold increase in CM number between 1 and 20 years of age (Mollova et al., 2013). Other cell type numbers change dynamically over time, with a reported 6.5-fold increase in endothelial cells and an 8.2-fold increase for mesenchymal cells (including fibroblasts) during heart growth. Interactions between these cells are multidirectional and exert great influence over crucial aspects of cardiac biology. Both endothelial cells and fibroblasts are key for tissue function and homeostasis. Aside from delivering oxygen and nutrients to the metabolically demanding CMs, the endothelium is fundamental for tissue hypertrophy and post-disease remodelling, (Holopainen et al., 2015) and maturation, (Giacomelli et al., 2020) displaying a strong paracrine influence (reviewed in Leucker and Jones, 2014). Fibroblasts also affect organ function and cell maturity, (Woodall et al., 2016; Wang Y. et al., 2020) whilst other cell types, such as immune cells, have been reported to display direct and significant interactions with CMs, as is the case with the electrical coupling of macrophages with atrioventricular node cells (Hulsmans et al., 2017). All in all, the general consensus supported by unambiguous histological evidence is that CMs are the largest fraction by volume, each nurtured by a median of 3 capillaries, where fibroblasts constantly keep the ECM through a degradation-deposition equilibrium. This brings us to our next player: the cardiac ECM.

#### ECM Composition

The cardiac cell types discussed above are arranged within a glycoprotein matrix which supports and provides them with a structure. Moreover, the cardiac ECM also has an active role in transmitting contraction and avoiding hyper-stretching of CMs. Its principal component is collagen, which accounts for 2–5% of the total weight of the heart, mainly types I (89%) and III (11%). Collagen type IV is present in the basement membranes, and collagen type V is located in the pericellular space (Weber, 1989; Eghbali and Weber, 1990; Sommer et al., 2015a). The collagen matrix has classically been categorised depending on which elements it tethers together into endomysium (binds adjoining CMs), perimysium (aggregates myocytes into myofibrils) and epimysium (present at the epicardial and endocardial surfaces). Cardiac fibroblasts have been identified as the main cell type responsible for secreting and remodelling the collagen matrix, although CMs seem to contribute to collagen type IV deposition (Eghbali et al., 1988). Apart from the structural function, the collagen network makes an important contribution to the whole myocardial tensile properties (Fomovsky et al., 2010).

Another key element of the cardiac extracellular matrix is elastic fibres. These are composites, made of an elastin core surrounded by a myriad of microfibrils. They provide elastic properties, by stretching upon mechanical demand and going back to their original length once the load is removed. Hence the importance of elastic fibres in tissues which have to accommodate their structure, such as skin, arteries or lungs or the heart. However, although elastic fibres are paramount for the heart’s elasticity, other factors are known to have an influence upon it, namely the proportion of muscle bundles to fibrotic tissue, and the density of collagen crosslinking (Forrest and Jackson, 1971; Parmley et al., 1973). In fact, elastic fibres are found in most cases close to the collagenous network and in intimate association with it (Sato et al., 1983). Of note, mature elastic fibres show slight architectural differences depending on the tissue (Kielty et al., 2002). As aforementioned, elastin forms the core of elastic fibres. Unlike most matrix proteins, which undergo a constant/continuous deposition and turnover, in healthy conditions elastin is synthesised only until adolescence (Dubick et al., 1981; Burnett et al., 1982; Davidson et al., 1982; Myers et al., 1985; Sephel et al., 1987; Parks et al., 1988; Pollock et al., 1990; Ritz-Timme et al., 2003). Other fundamental components of the cardiac matrix include, to a lesser extent, laminin, fibronectin, proteoglycans and glycoproteins (Fan et al., 2012). Laminin molecules are part of the basement membrane and are thus in close contact with the cell, playing an active role in modulating cell behaviour, including migration, differentiation and phenotype stabilisation (Yap et al., 2019). Fibronectin, besides promoting cell attachment, acts as an ECM organiser and is involved in collagen deposition (Valiente-Alandi et al., 2018). However, all components are crucial for tissue integrity and function.

Many, if not all, cardiovascular diseases have repercussions for the cardiac ECM. The reverse is also true. For instance, infarction studies in pigs show that the collagenous network starts to become disarranged after just 20 min of coronary occlusion, whilst elastin begins to disappear after 40 min, and both components appear detached from the basement membrane after 120 min. The balance of collagens I and III has been widely studied, revealing a significant increase in type III collagen after myocardial infarction (MI) (Sato et al., 1983). Dilated cardiomyopathies, in which the shape of the cardiac cavity is abnormal, are at least partly related to aberrant collagen remodelling, with less thick collagen and thinner fibres, which results in weaker tensile properties, more muscle slippage and wall thinning (Weber et al., 1988; Weber, 1989). Ventricular hypertrophy consists of the thickening of the ventricular wall associated with some conditions like hypertension, and it is found together with overexpression of collagen in the form of interstitial fibrosis (Eghbali and Weber, 1990).

#### Cardiac Architecture

In most tissues, structure and function are closely intertwined, and the heart is no exception. However, certain aspects of this relationship are still under debate. The overall manner in which the heart contracts and pumps blood is known, as is the arrangement of the tissue microstructure. The gap lies in providing a theory that explains how the different architectural elements interact to produce the global behaviour. For example, there is an ongoing debate about whether the myocardium forms a single myocardial band, (Buckberg et al., 2015a, b) or the so-called myocardial mesh model is more accurate (MacIver et al., 2018a, b). The controversy has two aspects. On the one hand, there is no consensus on whether the basic functional unit is the CM, or groupings of this into bundles (groups of CMs), sheetlets (groups of bundles), sheets (groups of sheetlets) or even laminae (groups of sheets). On the other hand, although imaging techniques allow us to visualise phenomena across the whole myocardium, it is not feasible to ascertain the distinct contribution of the individual functional units to producing the global outcome. According to Buckberg et al., the CM can undergo six functional events: shortening, lengthening, narrowing, widening, twisting and uncoiling (Buckberg et al., 2015a, 2018). There are an estimated 2.5-10 billion cells (Bergmann et al., 2015) in the heart, each of them performing one or more of these six actions in the same or a different direction, and all we are able to see is the macroscopic effect: a torsion-contraction movement of the organ. Still under controversy, there are at least 7 proposed models to accurately describe cardiac architecture, (Gilbert et al., 2007) which is widely recognised to have a profound effect, whether at a mechanical (LeGrice et al., 1995; Zócalo et al., 2008) or electrical (Roberts et al., 1979; Taccardi et al., 2008) level. During disease, myocardial architecture is severely disarranged, leading to inefficient contraction (Roberts et al., 1987; Wickline et al., 1992). It is expected that the application of advanced technology like diffusion tensor MRI (DT-MRI), which can obtain highly detailed information on fibre architecture, will soon shed light on this debate (Scollan et al., 1998; Poveda et al., 2013).

**FIGURE 2:**
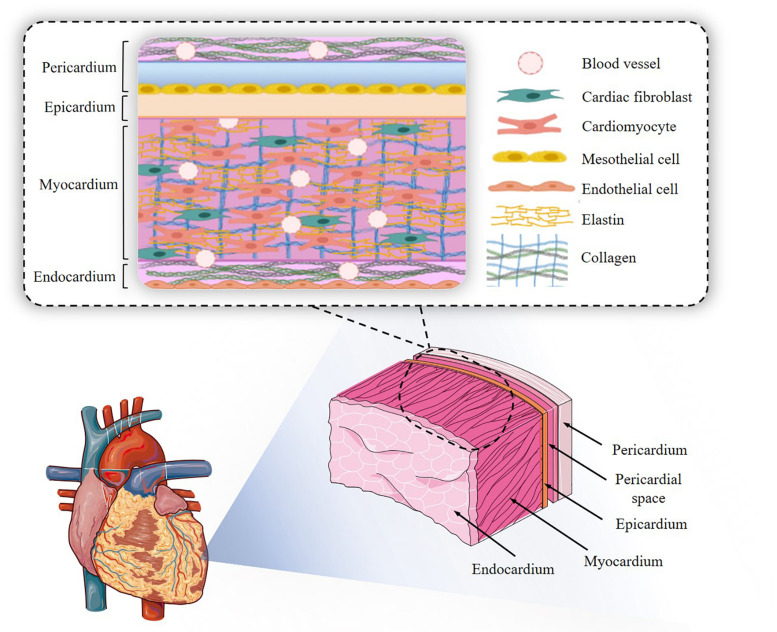
Cardiac structure. The endocardial-to-pericardial structure is outlined, with the main cellular and extracellular components.

At a simpler histological level, CMs (and CM bundles/sheetlets) are arranged in different orientations depending on their location in the organ, which in turn determines the direction of the stress produced. Myocytes are always in intimate contact with capillaries, which no doubt stems from the high metabolic demand of an ever-working muscular tissue: capillaries are located within 20 μm of CMs. Each CM is surrounded by a basement membrane containing laminin and collagen type IV, amongst others, and embedded in a highly structured ECM where collagen type I, as already discussed, is the main component (Figure 2). CMs connect to each other mainly by intercalated disks at their ends, but also through side branches, coupled to at least 2 CMs on the long axis and 1 laterally (Spach and Heidlage, 1995). Intercalated disks contain gap junctions, allowing fast current flow between neighbouring cells (Klabunde, 2012). As mentioned above, both individual CMs and groupings of these are surrounded by enveloping collagen. Fibroblasts do not participate in the electrical syncytium formed by the CMs, but rather lie in the interstitial space.

#### Cardiac Biophysical Properties

When attempting to engineer a tissue, it is essential to carefully recapitulate not only the cellular-extracellular components and their architecture, but also the resulting biophysical properties. These must reliably mimic those of their natural counterpart. In the heart, material properties are very complex: not only are they direction-dependent, but they also vary within the anatomical regions and the stage of the cardiac cycle. As an example, the literature reports variations in stiffness between the beginning and the end of diastole higher than an order of magnitude. For human LV, the reported values are 10-20 kPa at the beginning of the diastole, and 200-500 kPa at the end (Chen et al., 2008). Contraction itself results in a significant stiffening: from 0.5 to > 10 kPa. This magnitude is species-dependent, with a reported 3-fold increase in mouse, (Jacot et al., 2010) 2-fold in rat, (Prakash et al., 1999) and over 20 times in zebrafish (Krieg et al., 2008). Development also leads to a stiffening in the tissue, which arises from a relatively soft mesodermal layer (Krieg et al., 2008). Disease severely stiffens the organ, mostly due to the excessive deposition of collagen (scar for MI, interstitial in hypertension or other conditions), with values of over 50-100 kPa (Engler et al., 2008). Stiffness itself has a fundamental influence on how efficient CM contraction is, with CMs on softer- or stiffer-than-normal substrates doing little work or overstraining themselves, respectively (Engler et al., 2008). Reports on cardiac mechanical properties are extremely variable. This stems from a mix-up of animal vs human, fresh vs fixed, and healthy vs diseased data. In general, it is now accepted that most of the heart’s passive mechanical properties are due to the collagen in the matrix, (Sommer et al., 2015a) but at short sarcomere lengths the protein titin is the predominant contributor (Nguyen-Truong and Wang, 2018). Quoting Sommer et al., ‘results suggest that the passive human LV myocardium under quasi-static and dynamic multiaxial loadings is a non-linear, anisotropic (orthotropic), viscoelastic and history-dependent soft biological material undergoing large deformations’ (Sommer et al., 2015b). Or in simpler words: it is very complex and with multiple contributions from cellular/extracellular components. Added to this, scales differ, depending whether the tissue is macroscopically characterised using biaxial mechanical tests, (Sommer et al., 2015b) or whether isolated CMs are probed at a cell-relevant scale with Atomic Force Microscopy (AFM) (Andreu et al., 2014). Furthermore, some conflicting results have been reported, from reports showing force production from CMs to be increased with increasing stiffness, (Bhana et al., 2010) to stiffness having no influence at all (Jacot et al., 2008). In fact and as explained by Domian et al. (2017), it might even be the case that the material properties of cardiac tissue are not the main actor in the myocardial scenario, but this role is rather played by chamber pressure. More experimental and theoretical work needs to be done in this area before we reach a definitive conclusion.

Adding another layer of complexity, the heart has constant, potent and highly relevant electrical activity. Sparking at a small and specialised region called the sinoatrial node, the electrical wave travels through the auricles, reaching the atrioventricular node where it is delayed (allowing for the filling of the ventricles), and then spreads apex-to-base through the ventricles in a coordinated manner. All this process is controlled by a singular CM type, termed pacemaker cell, displaying disarrayed sarcomeres and low work generation capacity, but able to autonomously start the cardiac action potential. Ultimately, the action potential results in the entry of Ca^+2^ ions into the CM, releasing the sarcoplasmic stores of Ca^+2^ and freeing myosin of the inhibitory action of troponin I. CMs are also electrically connected through connexins, which form bridges between the cytoplasm of adjacent myocytes, effectively making the myocardium an electrical syncytium. However, it is an anisotropic one, with faster propagation in the direction of the fibres as opposed to the transverse direction (Chung et al., 2007). Conduction velocity is the speed with which the cardiac impulse travels from one point in the tissue to another. In adulthood, it lies in the range of 0.3-1 m/s, but developmental stage and disease will affect it (Yang et al., 2014). Achieving a similar value in any cardiac engineered tissue is paramount, given the fact that a mismatch between conduction velocities may give rise to potentially fatal electrical abnormalities such as arrhythmias (Kadota et al., 2013; Zhang et al., 2018). It is interesting that one of the foci in cTE is towards providing material-based electronic conductivity, although cardiac cells do not function by transmitting electrons but ions.

As mentioned already, both the mechanical and electrical properties exert a strong influence upon myocardial biology and function, in both health and disease. For example, increased fibrosis due to pathological conditions like MI or hypertension significantly stiffens cardiac muscle and affects CM contraction (Sessions and Engler, 2016). Ventricle loading induces CM elongation which, as explained by the Frank-Starling law, renders higher stroke with increase diastolic filling (Solaro, 2007). The coordinated conduction of the depolarisation wave throughout the organ, including the atrioventricular delay and the apex-to-base transmission, all contribute to optimal functionality and must be taken into account when engineering a human myocardium.

## Engineering Cardiac Tissue: The Building Blocks

cTE aims to generate tissue surrogates, either micro or macro, for various purposes, from developmental biology, (Young and Engler, 2011) to therapy (Miyagawa et al., 2018). In the following paragraphs, we will outline the main cells and materials, as well as the different fabrication technologies assayed in the field and the procedures for their maturation. Table 2 summarises some of the most relevant engineered myocardium examples, with a focus on human cardiac tissue.

**TABLE 2:** Summary of materials, cells and methods employed to engineer cardiac tissues, their biomimicry and resulting outcome.

REF	Materials		Fabrication	Cellular mimicry				Material mimicry			Maturation		Benefit of the selected approach?			
						
	Hydrogel	Fibres		CM	EC	SMC	CF	Mech.	Elect.	Align	Mech	Elec	vs.	Gene exp.	Structure	Function
Godier-Furnémont et al., 2015	Col I	–	Mould casting	Rat neonatal				Yes	No	No	+	+	NM	nd	+	+
Hirt et al., 2014	Fibrin	.	Mould casting	Rat neonatal				Yes	No	No	+	+	EHT	+	+	+
Jackman et al., 2018	FGN	–	Mould casting	Rat neonatal				Yes	No	No	+	–	NM	+	+	+
Amdursky et al., 2018	Albumin	–	Mould casting	Rat neonatal				Yes	No	No	–	–	2D	+	+	–
Nunes et al., 2013	Col I	–	Mould casting	hPSC				+	–	–	–	+	NM	+	+	+
Ruan et al., 2016	Col I	–	Mould casting	hPSC				+	–	–	+	+	EHT	+	+	+
Tiburcy et al., 2017	Col I	–	Mould casting	hPSC			FK	+	–	–	+	–	2D	nd	+	+
Valls-Margarit et al., 2019	Col I + ELN	–	Mould casting	hPSC			FK	+	–	–	+	+	2D	nd	+	+
Zhang et al., 2013	Fibrin	–	Mould casting	hPSC				nd	–	–	–	–	2D	+	+	+
Hirt et al., 2014	Fibrin	.	Mould casting	hPSC				+	–	–	+	+	EHT	+	+	+
Weinberger et al., 2016	Fibrin	–	Mould casting	hPSC	hPSC			+	–	–	+	–	NM	nd	–	–
Ulmer et al., 2018	Fibrin	–	Mould casting	hPSC				+	–	–	+	–	2D	–	+	nd
Ronaldson-Bouchard et al., 2018	Fibrin	–	Mould casting	hPSC			DF	+	–	–	+	+	2D	+	nd	nd
Jackman et al., 2016	FGN	–	Mould casting	hPSC				+	–	–	+	–	NM	nd	+	+
Shadrin et al., 2017	FGN	–	Mould casting	hPSC	hPSC	hPSC		+	–	–	+	–	NM	+	–	+
Dattola et al., 2019	PVA*	–	Foaming + FD	hPSC				+	–	–	–	–	2D	nd	–	nd
Han et al., 2016	–	PCL	SE	hPSC				–	–	+	–	–	2D	+	+	–
Joanne et al., 2016	–	Col I	SE	hPSC				+	–	–	–	–	NM	nd	+	+
Sireesha et al., 2015	–	POCS–FGN	SE	hCM				+	–	–	–	–	2D	nd	+	nd
Khan et al., 2015	–	PLGA	SE	hPSC				–	–	+	–	–	2D	–	+	–
Roshanbinfar et al., 2020	–	Col I/HA/PANi	SE	hPSC				+	+	–	–	–	EHT	nd	+	+
Macqueen et al., 2018	–	PCL/Gelat	Pull spinning	hPSC				+	–	+	–	–	2D	nd	+	+
Castilho et al., 2018	Col I	PCL	MEW	hPSC				–	–	–	–	–	2D	+	+	nd
Vaithilingam et al., 2019	PETra + MWCNT		3DP–SLA	hPSC				No	+	+	–	–	2D	nd	+	nd
Lee et al., 2019	Col I	–	3DbioP	hPSC			CF	+	–	–	–	–	NM	nd	+	–
Maiullari et al., 2018	FGN/PEG	–	3DbioP	hPSC	HUVEC			nd	–	–	–	–	EHT	nd	+	nd
Noor et al., 2019	dECM	–	3DbioP	hPSC	hPSC			+	–	–	–	–	NM	nd	+	+
Arai et al., 2018	–	–	3DbioP	hPSC	HUVEC		DF	nd	–	–	–	–	EHT	nd	+	nd

*REF, Reference; CM, Cardiomyocyte; EC, Endothelial cell; SMC, Smooth muscle cell; CF, Cardiac fibroblast; Mech, mechanical; Elec, electrical; Gene exp., Gene expression; Col I, collagen type I; FGN, Fibrinogen; hPSC, human pluripotent stem cell; FK, Foreskin fibroblast; DF, Dermal fibroblast; PVA, poly-vinil-alcohol; FD, Freeze-drying; ^∗^, foam; SE, Solution electrospinning; POCS, poly[1,8-octanediol-co-(citric acid)-co-(sebacic acid)]; hCM, primary human CM; PLGA, polylactide-co-glycolide; PCL, polycaprolactone; Gelat, Gelatin; PETrA, pentaerythritol triacrylate; MWCNT, Multi-walled carbon nanotubes; 3DP,3D Printing; SLA, Stereolithography; PEG, polyethylene glycol; ALG, Alginate; 3DbioP, 3D bioprinting; nd, not described; dECM, decellularised extracellular matrix; ELN, elastin; HA, Hyaluronic acid; PANi, polyaniline; NM, native myocardium; EHT, engineered heart tissue.*

### Cells

**FIGURE 3:**
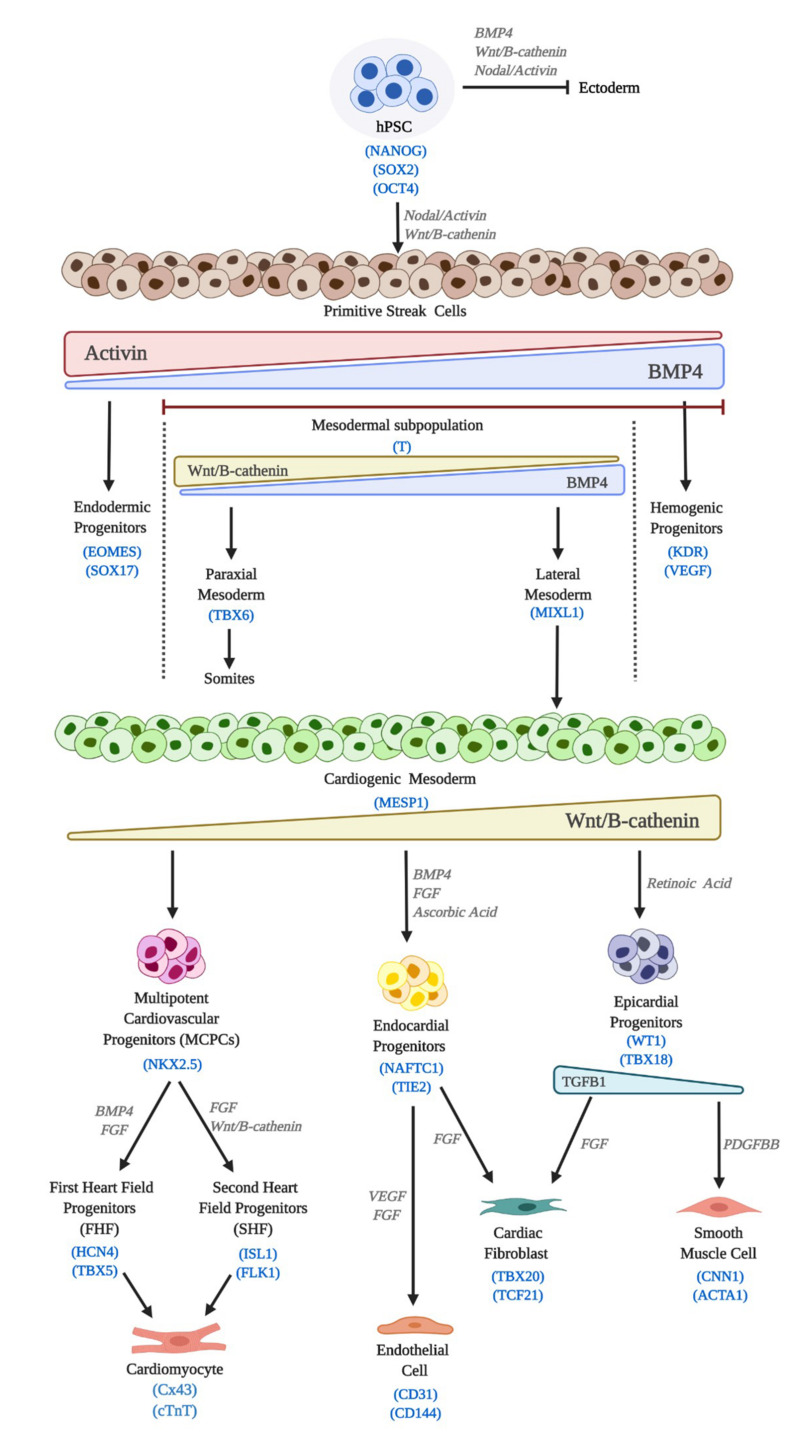
Cardiac differentiation of hPSC. hPSC differentiation *in vitro* mimics embryonic development. Induction signals, main molecular pathways and lineage markers are outlined.

The capacity to obtain human cardiac cell phenotypes in the laboratory began with the derivation of human embryonic stem cells (hESC) by Thomson and colleagues in 1998, (Thomson et al., 1998) which was soon followed by the first protocols for differentiation towards CMs (Mummery et al., 2003; Kattman et al., 2006). In 2006, thanks to the breakthrough of the reprogramming technology (Takahashi and Yamanaka, 2006; Yu et al., 2007) it became possible to relieve the field of some of its most notorious encumbrances, including the ethical ones. Both, hESCs and human induced pluripotent stem cells (hiPSC) fall within the wider category of human pluripotent stem cells (hPSC). Current methods, including scaling up protocols, (Serra et al., 2011) have paved the way for their widespread use. In general direct, efficient and reproducible hPSCs differentiation methods try to recapitulate embryonic development, from the induction of cardiac mesoderm, to CM, endothelial cells (ECs), cardiac fibroblast (CFs) or smooth muscle cells (SMCs) *in vitro* specification and maturation (Figure 3) (Burridge et al., 2015).

According to the culture format employed, derivation of CMs, CFs, ECs and pericytes/SMCs from hPSCs can be categorised into 3 main approaches: (i) inductive co-culture with visceral endodermal-like cells, (ii) suspension aggregates such as three dimensional (3D) embryoid bodies (EBs) and (iii) two-dimensional (2D) cell monolayer differentiation (Mummery et al., 2003; Kattman et al., 2006; Laflamme et al., 2007; Moretti et al., 2010). Early reports showed that co-culturing hPSCs with the mouse endodermal cell line END2 was able to induce beating foci (MacIver et al., 2018a). The low efficiency of this method, as well as the need for xenogenic co-culture, precluded its widespread application. EBs are formed by culturing dissociated hPSC in non-adherent plastic dishes and partially recapitulate the 3D structure and interactions of a developing embryo. hESC-EBs differentiate to derivatives of the three primary germ layers, resulting in spontaneously contracting outgrowths of human CM (Kehat et al., 2001). Based on EB differentiation protocols, CM from a variety of hESC and hiPSC lines have been generated, usually with a purity of < 10% (Zhang et al., 2009). ECs can also be isolated from spontaneously differentiating EBs, at a similarly low yield (≈2%) (Levenberg et al., 2002). In both cases, early reports explored the addition of cardiac mesoderm-inducing growth factors, including FGF2, VEGF BMP4, Activin A, Wnt agonists (WNT3A) or antagonists (DKK1), amongst others (Yuasa et al., 2005; Kattman et al., 2006, 2011; Yang et al., 2008; Tran et al., 2009; James et al., 2010). In general, however, EB-based differentiations have lost ground to more advanced and defined procedures, as the former are generally inefficient and render a mixture of cardiac cells with other non-cardiac phenotypes, requiring additional purification.

Monolayer-based differentiation is nowadays the most usually applied method. Cytokine-based protocols were developed first (Taccardi et al., 2008). These have been progressively modified by the discovery of Wnt signals playing a biphasic role in cardiac differentiation *in vivo*, (Marvin et al., 2001; Ueno et al., 2007) with early signals directing hPSCs towards cardiac fate, whilst later inhibition of those signals is a prerequisite for CM specification. Almost 10 years ago, this concept was incorporated into the CM differentiation from hPSCs, (Lian et al., 2012) paving the way for the grounding of a chemically defined procedure (Burridge et al., 2014). Based on small molecules rather than cytokines, and thus less costly, this protocol is now widely applied, providing highly pure yields of hPSC-derived CMs when in combination with a metabolic-based selection (Tohyama et al., 2013). This means that complicated and inefficient EB-forming procedures or expensive and time-consuming immune-selection protocols have now been discarded (Burridge et al., 2007; Hattori et al., 2010; Elliott et al., 2011; Uosaki et al., 2011). However, even this latest protocol still requires a degree of set up to avoid inconsistent efficiencies amongst cell lines and experimental repeats, mostly related to different patterns of endogenous early canonical Wnt expression (Paige et al., 2010). In general, CMs obtained from these protocols consist of a mixture of pacemaker, atrial and ventricular myocytes, though some researchers consider that this is open to question, as hPSC-CMs are immature and intrinsically plastic (Du et al., 2015).

The derivation of other cardiac phenotypes has been also achieved, with a variety of protocols now available. Palpant et al. reported the generation of CMs, cardiac- or hemogenic-derived ECs as well as blood cells by finely dosing BMP4 and Activin A in order to pattern hPSC towards different mesodermal fates (Palpant et al., 2017). Others have employed a mixture of small molecules and cytokines to derive vascular cells from hPSCs in monolayer culture with high efficiency (Orlova et al., 2014; Patsch et al., 2015). Global gene transcription analysis has demonstrated low variability between ECs differentiated via cytokine-based methods from multiple lines of hPSCs (White et al., 2013). CFs, have been increasingly recognised as major players in cardiac development and homeostasis, having a similarly significant effect upon the capacity to build cardiac tissues in the lab. Recently, two independent groups have reported the generation of hPSC-derived CFs, giving also proof of their capacity to affect hPSC-CM function (Zhang H. et al., 2019; Zhang J. et al., 2019). Epicardial cells have similarly been derived, (Witty et al., 2014) demonstrating their ability to increase the therapeutic capacity of hPSC-CMs *in vivo* (Bargehr et al., 2019). Finally, sinoatrial node pacemaker CMs have been obtained from hPSC, and their capacity to pace tissues *in vivo* has been reported (Protze et al., 2017). Other approaches to the differentiation of cardiac lineages include the generation of CVPs, (Blin et al., 2010; Birket et al., 2015; Zhang Y. et al., 2016) or direct reprogramming strategies, (Mohamed et al., 2017) but they have rarely been explored in cTE.

### Materials

In parallel to the way differentiation of hPSC mimics the natural embryonic development, the current view is that the more a material replicates the properties of cardiac tissue, the higher the chances of success. Development over the last 15 years has yielded a wide portfolio of materials and biomaterials. Classifications are numerous, be it by origin (natural, synthetic or hybrid), crosslinking (chemical vs physical), size (macro, micro or nano), polymerisation mechanism (enzymatic, light-triggered or pH-responsive) or whether they are or not reinforced with other structures like fibres. For specific insight into these classifications, we direct the reader towards some of the excellent latest papers (Peña et al., 2018; Liu et al., 2019; Xu et al., 2019). One of the most relevant classifications is, however, on the physical consistency of the applied material, where we can differentiate (i) injectable materials and hydrogels, (ii) solid or fibrous scaffolds and (iii) composite systems.

Hydrogels are probably the most widely explored type of material in cTE. Collagen, being the main component of the cardiac ECM, has been widely employed (see the following sections). It can be readily isolated from animal or even human tissues in sufficient quantities, extracted and solubilised, although it requires acidic pH for this. Therefore, a careful control over pH is needed for optimal polymerisation and embedded cell survival. Gelatin, being denatured/digested collagen, has also been intensely explored and the basis for the generation of some of the most applied semi-synthetic materials, such as gelatin methacryloyl (GelMA)(Yue et al., 2015) and several biorthogonal derivatives (Koshy et al., 2016; Bertlein et al., 2017). Alginate, a sugar-based natural hydrogel obtained from algae, (Orive et al., 2006) has been employed due to their tailorable mechanical properties and simple polymerisation, mediated by cations such as Ca or Mg, albeit lacking biological binding motifs. Silk and its derivatives have also been processed into hydrogels, (Holland et al., 2019) and modified to incorporate electrically active particles (Barreiro et al., 2019) or photocrosslinkable chemical groups (Cui et al., 2020). Allergic and anti-inflammatory side reactions have been reported with some silk derivatives, so care should be taken when incorporating them into an engineered tissue. Amongst natural hydrogels, decellularised ECM (dECM) has attracted significant interest since the breakthrough discovery of the process, as applied to the building of tissues (Ott et al., 2008; Belviso et al., 2020). In principle, dECM retains all components of the ECM of origin, thus creating a more complex and biomimetic environment. Garreta et al. obtained human dECM slices on which they cultured hPSC-CMs. The human CMs demonstrated enhanced conduction velocity and gene expression of related genes (SERCA, KCNJ2, CACNA1C or SCN5A amongst others) (Garreta et al., 2016). The group of Lior Gepstein employed dECM-chitosan mixtures in combination with hiPSC-CMs. The resulting engineered myocardium displayed enhanced maturity as compared with cells cultured in 2D, showing tissue-like drug responses. Their work also provided proof-of-concept of the capacity of this system to model human cardiac diseases (Long QT syndrome) and arrhythmias (Goldfracht et al., 2019). On the synthetic side, polyethylene glycol (PEG) and its several modifications have been extensively studied in the TE field (Iyer et al., 2009).

Most of the materials so far outlined in this section can be processed into fibres employing strategies explained in the next section. Thermoplastics have also been applied to cTE, mostly as fibrous scaffolds. Examples include poly-ε-caprolactone (PCL), (Woodruff and Hutmacher, 2010) or elastomers like poly (glycerol-sebacate) (PGS) (Kharaziha et al., 2013). Conductive polymers have only recently began to be applied to the field, though some of the most remarkable examples have not incorporated the use of cells and would therefore not qualify as engineered tissues (Mawad et al., 2016; Kapnisi et al., 2018). Finally, given the low mechanical properties displayed by most hydrogels, composite fibre-reinforced materials are also being developed, (Bas et al., 2015) with some examples explained in the following section.

### Maturation Stimuli

Cells derived from hPSC are immature (see Karbassi et al., 2020 for a review). Although not the direct focus of this work, neonatal myocytes, which are another cell source commonly employed, also suffer from this drawback. cTE has long been aware of this limitation and has applied three main stimuli, namely physical, mechanical and electrical, and combinations thereof Parsa et al. (2016) and Stoppel et al. (2016). Perfusion is able to improve engineered tissues’ properties, as it will boost nutrient access and renewal, as has been shown for neonatal cells (Radisic et al., 2004b) as well as hiPSC-cardiac derivatives (Valls-Margarit et al., 2019). Materials’ physical properties (e.g., stiffness) are able to induce maturation features in these CMs, or at least preserve primary CMs from dedifferentiation (Amdursky et al., 2018). In general, most hydrogels are able to replicate the right myocardial-like properties. For example, Feaster and colleagues found that plating hiPSC-CMs on thick Matrigel induced a certain degree of molecular and functional maturation, in comparison to thin, diluted hydrogel coating, which essentially transmits the rigidity of the underlying plastic (Feaster et al., 2015). Herron et al. employed soft (albeit supra-cardiac) substrates and compared them with glass in their capacity to influence hiPSC-CMs, with a significant effect on maturation, showing improvement in the expression of Na and K channels, as well as on the degree of binucleation, cell cycle exit and hypertrophy (Herron et al., 2016). Although not purely based on engineered tissues, they and others give proof of the crucial role stiffness plays in cardiac maturation.

Mechanical stimulation has a leading role in cardiac development and aging (Happe and Engler, 2016; Sessions and Engler, 2016). It can be isometric, isotonic or auxotonic (Liaw and Zimmermann, 2016). In isometric stimulation, the construct is preloaded and must exert force against a static load. In isotonic stimulation, a device will cyclically exert active elongation on the engineered tissue. Finally, auxotonic stimulation occurs when the myocardial tissue has to contract against a resilient load. In principle, the auxotonic mode confers a more physiological stimulation, although all three are reported to deliver maturation upon engineered myocardium (Godier-Furnémont et al., 2015; Lux et al., 2016; Ruan et al., 2016; Ulmer et al., 2018). Although the exact mechanisms by which mechanical stimulation matures the cardiac engineered tissue are not known in depth, it is presumed that they will operate by the same ones occurring during cardiac development or physiologic hypertrophy (Nakamura and Sadoshima, 2018). After all, being a striated muscle, the myocardium can undergo hypertrophy if exercised (Kim et al., 2008).

As an electro-sensitive organ, the heart can be stimulated by electrical pulses, which can help maintain its function *ex vivo* (Watson et al., 2019). Electrical stimulation is known to have a relevant effect on hPSC-CM differentiation and maturation. Over 20 years ago, Sauer et al. established a relationship between this stimulation and mouse ESC differentiation to CM (EB method). They showed the effect was at least partly mediated by reactive oxygen species (ROS) generation and NF-κB, and could be replicated by ROS from H_2_O_2_ incubation (Sauer et al., 1999). Serena et al. analysed the effects of the type of electrode and stimulation length on CM differentiation via the EB method from hESC, finding a role for ROS, though the effect on the efficiency of differentiation was not determined (Serena et al., 2009). Hernández et al. employed brief (5 min) electrical stimulation, of hiPSC-EBs, finding an increase in the percentage of cardiac differentiation (% of beating EBs) after 14 days (Hernández et al., 2016). However, the use of the EB method complicates findings, as the effect could also be mediated by other cell types within the EB. Electrical stimulation has also been shown to enhance hPSC-CM maturation at the gene expression and functional levels (Ca transients), as well as promoting the ventricular phenotype (Chan Y. C. et al., 2013). Reasoning that exogenous electrical stimulation would act as an artificial pacemaker, Richards and coworkers evaluated the implementation of electrically conductive silicon nanowires in hiPSC-derived cardiac spheroids, showing that it was able to improve CM-to-CM communication (measured by staining for Connexin 43 and N-cadherin) and structural quality, though some of these quantifications might nowadays be regarded as debatable (Richards et al., 2016). The cTE field has implemented electrical stimulation to cardiac constructs with success. The group of Gordana Vunjak-Novakovic pioneered work in this area, showing the enhanced of contraction (synchronicity) and structure (alignment, ultrastructure) on neonatal rat CMs seeded on Ultrafoam collagen sponges and Matrigel (Radisic et al., 2004a). The group developed stimulation protocols as well as bioreactors, (Tandon et al., 2008, 2009; Massai et al., 2013) which have influenced the whole field. The group of Milica Rasidic built hPSC-based cardiac tissues by embedding hPSC-dissociated EBs in collagen type I and Matrigel. After electrical stimulation, they showed an increased myofibril ultrastructural organisation and improved function (conduction velocity and Ca handling properties) as compared to non-stimulated controls (Nunes et al., 2013).

Finally, some remarkable advances have been made when combining electrical and mechanical stimulation. Ruan et al. generated collagen-based cardiac engineered tissues containing hPSC-CMs, which were subjected to electromechanical stimulation and compared to static stretch or no stimulus. Results showed a positive Frank-Starling effect (increased force production with increased preload), a less negative force-frequency relationship (increased force production with increased pacing frequency) and maximum stress generation for the electromechanical stimulation group, which was correlated with increased expression of RYR2 and SERCA2, thus supporting the use of combined stimulation for enhanced maturation (Ruan et al., 2016). The group of Dr. Zimmermann employed auxotonic stimulation delivered through stretchers in combination with electrical pacing at 0, 2, 4, or 6 Hz (Godier-Furnémont et al., 2015). Results on tissues generated with collagen and neonatal rat cells showed that the 4 Hz regime was able to generate tissues with a physiological and positive force-frequency and enhanced functionality. Also, the presence of T-tubules was demonstrated. Finally, the group of Prof Vunjak-Novakovic generated hiPSC-CM-based collagen tissues on flexible stretchers (auxotonic mechanical stimulation) and supplied no stimulation (control), 2 Hz and 0.33 Hz/day progressive increase over 2 weeks (‘intensity training’). Their results demonstrated the effectiveness of this strategy, as shown by a physiological sarcomere length, increased density of mitochondria, T-tubules, a more mature metabolism and functional improvements at the level of Ca cycling and a positive force-frequency relationship (Ronaldson-Bouchard et al., 2018).

### Fabrication Strategies

Materials confer cTE with significant options, not only due to the available range, but also through the application of different fabrication modalities, which can deliver different properties out of the same starting material. For example, collagen can be mould-casted, (Godier-Furnémont et al., 2015) extruded, (Araña et al., 2014) or bioprinted, (Lee et al., 2019) and the resulting properties will vary widely, with casted and bioprinted collagen having a stiffness in the range of kPa, whilst the extruded film will be significantly stiffer (MPa). In addition, structure will also differ. 3D printing and bioprinting have no doubt revolutionised our capacity to engineer cardiac tissues, however, other technologies can provide relevant features. The following paragraphs outline some of the most employed strategies, classified depending on their capacity to produce a controlled architecture (Table 3).

**TABLE 3:** Summary of fabrication methods, advantages and disadvantages.

		Fabrication technique	Advantages	Disadvantages
No true architecture control	Mould casting	Mould casting (Janik and Marzec, 2015)	– Simplicity and cost – High cell survival – Range of compatible materials – Mechanical properties – Scale up feasible	– Lowest architectural control – Limited thickness
	Pore-forming	Solvent Casting, Particle Leaching, Cryogelation (Janik and Marzec, 2015)	– Control of pore size – No specialised equipment – Cost	– Use of organic solvents – Limited scaffold thickness – Mechanical properties – Time consuming leaching – Limited pore architecture
	Electrically produced	SE (Liang et al., 2007)	– Nanometer features – Range of compatible materials – Scaling up feasible	– Use of organic solvents – Limited scaffold thickness – Usually produces high stiffness substrates
	Textile- based	Weaving, Braiding, Knitting (Akbari et al., 2016)	– Simple – Scaling up feasible	– Specialised equipment (cost) – Not widespread – Limited porosity
True architecture control	Build & seed	SLA (Melchels et al., 2010)	– High architectural control – Self-supporting process	– Only photosensitive polymers – Remove supporting materials – Use of UV light – Toxicity of photoinitiator – Specialised equipment (cost)
		SLS (Mazzoli, 2013)	– No solvents required – High architectural control – Self-supporting process – Range of compatible materials	– High temperature – Materials in powder form – Rough surface – Specialised equipment (cost)
		MEW (Brown et al., 2011)	– No solvents required – Control over porosity, pore size and fibre diameter – High architectural control	– Limited thickness – Range of available material – Specialised equipment (cost)
		FDM (Moroni et al., 2006)	– No solvents required – Speed of printing – Good reproducibility	– Restricted to materials with good melt viscosity properties – High temperature – Filament required – Limited resolution
	Bioprinting	LGDW, LIFT, BioLP (Koch et al., 2010; Gaebel et al., 2011; Hu et al., 2017)	– Range of cells/biomaterials – Single cell resolution – Precise cell printing	– Low cell viability – Limited 3D structure – Time consuming
		3DbioP (Zhang et al., 2015)	– Range of cells/biomaterials – High cell viability – Process at room temperature	– Weak structural support – Specialised equipment – Multimaterial printing expensive (multinozzle)

*SE, Solution electrospinning; LGDW, Laser-guided direct writing; LIFT, Laser Induced Forward Transfer, BioLP, Bio-laser Printing; 3DbioP, 3D bioprinting; SLA, Stereolithography; SLS, Selective laser sintering; MEW, Melt Electrospinning Writing; FDM, Fused deposition modelling.*

#### Techniques With No True Architecture Control

##### Mould casting

Probably the simplest and most widely employed fabrication mode, requires the generation of a mould of the desired shape, and is the fabrication technique of choice in many biomedical-based laboratories, where other methods could not be implemented due to lack of expertise or specific equipment. It does not provide much control over the resulting architecture, but can be combined with others, like porogen leaching, to add specifically selected features to the resulting tissue. The first reports on engineered cardiac tissues by Thomas Eschenhagen and coworkers in the late 1990s were developed by mould-casting a mixture of chick embryonic CMs embedded in a collagen solution. This was allowed to gel between two Velcro-coated glass tubes. The resulting tissues, later termed engineered heart tissues (EHTs), could be cultured *in vitro* and maintained over several days, responded to electrical stimulation, displayed a positive Frank-Starling relationship, were sensitive to levels of extracellular calcium, and could be modified with viral vectors (Eschenhagen et al., 1997; Zimmermann et al., 2000).

Since then, the technology has evolved enormously. It was expanded to neonatal rat cells, (Zimmermann et al., 2002) and refined, with the possibility of fabricating more complex and thicker EHTs, which functioned synchronously (Zimmermann et al., 2006). These stacked EHTs also showed promise as a therapy when transplanted in a rat model of infarction. EHTs have been employed by the groups of Eschenhagen and Zimmermann, either fibrin- or collagen type I-based, to study the effect of chronic stretch on CM hyperthrophy, (Fink et al., 2000) as a disease model of hypertrophic cardiomyopathy, (Stöhr et al., 2013) or to analyze the effect of electrical stimulation (Hirt et al., 2014). EHT technology has greatly benefited from the implementation of hPSC-derived cells, as the findings, models and application have gained greater impact, be it as a drug testing platform (Eder et al., 2016), an *in vitro* tool to study cardiac stimulation, (Godier-Furnémont et al., 2015) or as a potential myocardial regenerative therapeutic (Weinberger et al., 2016; Tiburcy et al., 2017). The Bursac group has also employed mould casting of rat and human (hiPSC-derived) cells to engineer cardiac tissues and explore different strategies to mature them *in vitro* (Jackman et al., 2016). They applied this strategy to fabricate tissues up to human scale, though thickness was limited by nutrient and oxygen diffusion. When assayed in a rat model of disease, their cardiopatches retained integrity after 3 weeks, showing extensive vascularisation by host-derived vessels and maintaining electrical activity, although they did not functionally integrate with the endogenous myocardium (Shadrin et al., 2017). The team of Milica Radisic employed PDMS moulds to form engineered cardiac tissues around a surgical suture, composed of hPSC-derived cardiac cells, collagen type I and 10% Matrigel (Nunes et al., 2013). By employing electrical stimulation, through carbon rods immersed in the culture medium, they were able to drive the hPSC-CMs towards a more mature phenotype and functionality. This system was later applied also to drug testing (Feric et al., 2019). Finally, one of the most advanced pieces of evidence for hPSC-CM maturation *in vitro* was provided by the group of Gordana Vunjak-Novakovic, as explained in the previous section (Ronaldson-Bouchard et al., 2018). They employed a 3:1 mixture of hPSC-CMs and dermal fibroblasts embedded in a fibrin matrix, cast into wells where flexible PDMS posts were also included. These conferred pre-tension on the generated engineered myocardium, thus adding physiological-like auxotonic stimulation (Mannhardt et al., 2019). A set of custom-made carbon electrodes delivered electrical stimulation. The resulting bioartifical myocardium displayed adult-like gene expression profiles, ultrastructural features such as M-bands and T-tubules, as well as oxidative metabolism and mature functionality. In summary, although mould casting cannot generate fine features, it is one of the most widely applied and highly evolved methods to obtain human mature cardiac tissue, and the one closest to translation.

##### Macro-to-micro pore-forming strategies

Cryogelation, based on freeze drying, is a common preservation strategy that is also applied to TE. The pre-crosslinked material is subjected to freeze-thawing cycles, which induces ice crystal formation. These crystals act as porogens, to be removed when pressure is decreased and the solvent sublimated. In general, pores are well inter-connected, as demonstrated early on by O’Brien and colleagues for collagen-glycosaminoglycans scaffolds (O’Brien et al., 2004). In addition, varying conditions make it possible to modulate material properties, as shown by Kim et al. (2015). In combination with gas foaming, Dattola et al. fabricated a poly(vinyl) alcohol scaffold with pores within the size of CMs, and Young’s Modulus similar to that of the native cardiac ECM. These substrates are able to support the growth and cardiac differentiation of hiPSC, albeit at apparently low rates as demonstrated by the immunostaining for cardiac proteins (Dattola et al., 2019).

Gas foaming has also been extensively applied to TE, though its use within the cardiac field is not extensive. This technique relies on the formation of bubbles, either by adding exogenous agents such as sodium bicarbonate or through the inclusion of enzymatic-driven reaction in the fabrication process (Mooney et al., 1996). As the solution polymerises, these bubbles are trapped inside. In consequence, the choice of foaming agent is crucial for the later survival of cells. Although relatively simple to implement, this technique provides no control over the degree of interconnection between the formed pores or their directionality.

Porogen templating is based on the inclusion of salt crystals in the pre-polymer solution. Similar to freeze drying, these crystals will act as templates, creating an empty space (pore) when the particles are leached out, in most cases by dissolving in aqueous solution, weak bases or heating, (Thomson et al., 1995) with sodium chloride being one of the most frequently employed porogens. On the down side, limitations are related to low architectural control and suboptimal processing of porogens, which might compromise biocompatibility as well as mechanical properties. Salt leaching has not been as widely employed in cTE as in other areas. Ganji et al. used table salt as a simple yet efficient way to generate defined pores in polyurethane-based scaffolds where gold nanotubes and nanowires were incorporated, thus combining 3-dimensionality with the additional benefit of electrical conductivity (Ganji et al., 2016). Biocompatibility tested with H9c2 rat cardiomyoblasts showed a positive effect on cell growth, though no hPSC-derived cells were tested. Other examples have employed porogen templating for the fabrication of porous polysaccharide-based vascular scaffolds and engineered heart valves (Lavergne et al., 2012; Masoumi et al., 2014).

Thermally induced phase separation is based on the use of temperature to induce the de-mixing of a homogeneous polymer solution. The controlled change in temperature prompts the formation of a polymer-rich and a polymer-poor phase, which can be used to obtain an interconnected porous structure (Nam and Park, 1999). Vozzi et al. synthesised the elastomer polyesterurethane, and used thermal phase separation to create a porous and biocompatible structure, which they functionalised with fibronectin by NHS-EDC chemistry. Although material properties were off the cardiac optimal range (in the order of > 0.25 MPa), neonatal rat cardiac cells attached and grew on the scaffolds, with specific modulation of gene expression. However, no use of human cells was reported (Vozzi et al., 2018).

##### Electrically produced

Solution electrospinning (SE), on the other hand, was one of the first and most employed fabrication modalities in the field. In this technique, a polymer solution is subjected to a relatively high electric field while it travels towards a collector plate. This electric field causes the polymeric jet to whip, producing very thin fibres of even sub-micron size, that can be collected randomly or in an aligned fashion. For a review see Sensini and Cristofolini (2018). In general, most SE applications produce low thickness mats of (semi-)randomly arranged fibres of different materials, though use of appliances such as a rotating mandrel can increase alignment (Han et al., 2016). Although relying on the use of potentially toxic solvents, SE has the advantage of being open to a wide range of materials, both natural and synthetic (Kitsara et al., 2017).

The concurrent use of SE with hPSC-derived cardiac cells has not been widely explored but some remarkable examples can be found. The group of Onnik Agbulut tested different crosslinking times of a clinical-grade collagen electrospun scaffold for the most suitable conditions for hiPSC-CM culture. The resulting mats had fibres of 0.6-2.2 μm, with pores in the range of 2-3 μm. After no deleterious effects were found after mat implantation in animals, the mats were seeded with hiPSC-CMs (10^6^ per scaffold), and produced a significant benefit when transplanted in a dilated cardiomyopathy model. Importantly, scaffolds were compliant enough to allow the generation of macroscopic contractions by the hiPSC-CMs (Joanne et al., 2016). Sireesha et al. used a combination of the elastomer poly[1,8-octanediol-co-(citric acid)-co-(sebacic acid)] and fibrinogen to produce scaffolds with a sub-micron fibre diameter, whose mechanical properties laid on the upper end of cardiac elasticity (hundreds of kPa) and which showed good biocompatibility with human CMs. However, no further tests in animal models were performed (Sireesha et al., 2015). Khan and co-workers electrospun polylactide-co-glycolide (PLGA) as 50 μm-thick aligned nanofibrous scaffolds, seeded with hiPSC-CM, and compared the outcome versus conventional tissue culture plastic surfaces. Results showed the scaffolds were able to align the CMs, as well as inducing changes at the level of functionality (Ca transients) and gene expression, though the high stiffness of the mats (1-2 orders of magnitude above the cardiac tissue) could have a negative impact (Khan et al., 2015). On the other side of the coin, Han et al. assayed the capacity of aligned electrospun mats made of PCL coated with matrigel to induce hiPSC-CM maturation, showing a limited effect at the functional level, but with some differences in gene expression (Han et al., 2016).

Aside from employing different materials or combinations of natural and synthetic polymers, SE offers increased possibilities by modifying the properties of the resulting engineered tissue by combination with nanoparticles or other fabrication technologies. For example, the group of Tal Dvir built on his previous work on the electrospinning of albumin (Fleischer et al., 2014) to increase the anisotropy of the fabricated engineered tissue. To do this, they employed a double strategy: they used laser patterning to create micro-holes and unidirectional grooves, in order to increase mass transport and cell alignment respectively, and they stacked several layers of patterned mats, inspired by the anisotropy found across the ventricular wall. In this fashion, they also modulated the mechanical properties of the layers, getting closer to human myocardial values. They did not, however, test the stiffness of the resulting stacked construct, nor populate it with human cells, using rat neonatal cardiac cells instead (Fleischer et al., 2017). The group of Ali Khademhosseini used poly(glycerol sebacate):gelatin (PG) solution where gelatin-methacryloyl (GelMA)-coated carbon nanotubes had been incorporated to electrospun aligned nanofibrous mats with improved electrical properties, though at the cost of increased stiffness (Kharaziha et al., 2014). This is similar to their previous work with GelMA-embedded carbon nanotubes, (Shin et al., 2013) with a significant effect on gene expression, structure and functionality, but again no human cells were employed. Walker et al. employed bio-ionic liquids to modulate the electrical properties and adhesion strength of electrospun GelMA scaffolds, assaying their *in vitro* and *in vivo* regenerative potential, though no tissue was constructed (Walker et al., 2019). Again, mechanical properties were affected. Recently, the group of Felix Engel used SE to generate fibres combining the conductive polymer polyaniline (PANi) with collagen/hyaluronic acid, producing mats of suitable mechanical and electrical properties. Scaffolds supported the attachment of hiPSC-CMs, which displayed typical striations and contractions. Substrates incorporating PANi induced a faster beating rate on hiPSC-CMs, though this was not further explored (Roshanbinfar et al., 2020). All in all, SE is a very versatile fabrication technique, and this is also increased by the possibility of combining it with other materials and technologies. However, it faces limitations related to the thickness and dimensions of the final construct, as well as long-range alignment capacity.

##### Textile-based fabrication

Historically speaking, weaving is one of the oldest fabrication techniques and many (bio)materials can be processed by knitting, weaving or braiding, (Akbari et al., 2016) giving rise to new architectures and importantly, mechanical properties unattainable for individual fibres. Curiously, these have not been mainstream in the cTE field, with few studies combining these fabrication methods with hPSC-derived cells. Though their work cannot fully be categorised as textile-based, the lab of Kevin K. Parker employed pull spinning to generate nanofibrous scaffolds composed of PCL/Gelatin over an ellipsoidal mandrel, mimicking the shape of an idealised ventricle. After additional coating with the ECM protein fibronectin, they seeded either neonatal cardiac rat cells or hiPSC-CMs, generating scale-models of the heart with striking functional properties (Macqueen et al., 2018).

#### Techniques With True Architecture Control

Additive manufacturing, also known as 3D printing, is one of the technological revolutions of this century. It is based on the building of a volumetric object from a computer aided design (CAD), often in a layer-by-layer basis. Broadly speaking, we can distinguish between bioprinting, where cells are also printed, from the build & seed strategies, where the scaffold is first printed and the cells incorporated in a subsequent step, either as a standalone element or embedded in a hydrogel matrix. In any case, the degree of control over the resulting structure is orders of magnitude above what is achievable with the above-mentioned technologies, with the exception of filament dimensions, with SE being able to go into sub-micron diameter, albeit at the expense of thickness and architecture.

##### 3D Build and Seed

In this category we have gathered those additive manufacturing technologies that do not allow the concurrent printing of materials and cells, be it because they employ high temperature, damaging lasers, toxic solvents or for other reasons. This section includes selective laser sintering (SLS), consisting of iteratively spreading layers of powdered materials and fusing it together to achieve the programmed shape, (Duan et al., 2010) stereolithography, where a bath with photosensitive resin is selectively cured layer by layer with a UV laser or similar power source, (Gauvin et al., 2012) or fused deposition modelling (FDM), by which the printed polymer is melted and deposited in layers in order to acquire the desired 3D architecture (Moroni et al., 2006). All these technologies have in common the need to first generate the architecture and in a second step add the biologicals, with the only exception of laser-curable materials in SLS. Although these technologies are in increased demand for the building of prosthetics, personalised solid implants or educational/surgery planning models, (Giannopoulos et al., 2016) their application to cTE is not widespread, especially in light of the increasing importance of bioprinting techniques (next section).

A special note however must be dedicated to Melt Electrowriting (MEW), a highly specialised additive manufacturing technology (Brown et al., 2011). It works on the same basis as SE, where a high voltage is applied between a syringe tip and a grounded collector. In this case however, the polymer is not dissolved, but melted. The superior stability of the jet allows a highly controlled deposition of fibres, which solidify in-flight or shortly upon collection. These, although bigger in diameter than those from SE, are at least an order of magnitude below what is usually attainable with 3D printing or bioprinting (tens of μm in diameter). The polymer of choice must be melted within the capacities of the printer, which limits the range of the available polymers. Another significant limitation is the achievable 3-dimensionality, which is usually below the mm-range. The groups of Paul Dalton and Dietmar Hutmacher have spearheaded its use (Bas et al., 2015, 2017; Hochleitner et al., 2018) and have developed methods to increase the thickness of the MEW scaffolds to almost one cm, but again these are far from mainstream (Wunner et al., 2018). The groups of Jos Malda and Joost Sluijter have been the first ones to apply MEW to cTE. In their first approach, they MEW-printed the hydroxyl-functionalised polyester, (poly(hydroxymethylglycolide-co-ε-caprolactone) in orthogonal patterns, and seeded them with human cardiac progenitor cells (CPC) embedded in a collagen type I hydrogel. CPCs survived well, with the scaffolds producing a significant alignment (Castilho et al., 2017). In a further optimisation of the system, Castilho et al. fabricated hexagonally patterned MEW scaffolds with superior compliance, and seeded them with hiPSC-CMs. Their results showed the superior biaxial mechanical properties of the hexagonal designs, and their significant, positive effect on cell alignment and gene expression. Moreover, these scaffolds could be transplanted in a large animal (pig) after passing through a catheter-like tube, paving the way for their future percutaneous transplantation (Castilho et al., 2018). Finally, an auxetic patch, that is, featuring a negative Poisson ratio, was recently fabricated with MEW, and the conductive polymer polypyrrole *in situ* polymerised on it, with electroconductive properties close to those of the human myocardium (Olvera et al., 2020).

##### Bioprinting

The capacity to print human tissues on-demand has roused the expectations of scientists, clinicians and patients alike, with a range of modalities available. Laser-assisted bioprinting (LAB) for example, features several methodologies. Laser-induced forward transfer of material/ink (LIFT)(Koch et al., 2010) allows printing with a single-cell resolution at high densities (up to 10^8^ cells/ml) (Gaebel et al., 2011). However, it requires selected materials to gel relatively fast, which in practice restricts the range of available materials, and can be technically challenging. 2 photon polymerisation is a type of LAB with high spatial resolution (as low as 70 nm) without the need for support structures (Hu et al., 2017). Vaithilingam et al. employed this technique to fabricate electroactive 3D architectures by incorporation of multi-walled carbon nanotubes (CNTs). The mechanical properties were however highly deviated from what has been described for human cardiac tissue (GPa range). hiPSC-CMs were able to survive and, after the application of electrical stimulation, a significant effect on sarcomere length was found (Vaithilingam et al., 2019). There are other printing technologies based on LAB, such as Biological Laser Printing and Laser Guided Direct Writing, (Odde and Renn, 2000; Barron et al., 2004) but no reports on these include hiPSC-derived cardiac cells.

Extrusion-based bioprinting is probably the best-known bioprinting method: a bioink containing both cells and the biomaterial is extruded through a nozzle onto a motorised collector. The coordination between the gelation of the bioink and its deposition permits the generation of thick tissues. A range of nozzles, including pneumatic, screw- or plunger-based, as well as inks, is available. However, the material properties of the latter are fundamental, as shear stress generated upon printing highly impacts cell viability (Jungst et al., 2016). Some strategies, like *in situ* crosslinking of the bioink immediately prior to its deposition, (Ouyang et al., 2017) are being developed to overcome these and other limitations. Extrusion-based bioprinting has been extensively researched in the cTE field. Jang et al. employed a multi-nozzle printer to generate tissues with 2 decellularised cardiac ECM-based bioinks reinforced with a thermoplastic (PCL) backbone. One of the bioinks contained human CPCs (c-kit-positive), and the other mesenchymal stem cells (MSC) and pro-angiogenic factors (VEGF). The application of this patch in a rat model of disease demonstrated a rapid vascularisation as well as a significant benefit at the functional and histological levels. However, no new contractile tissue was formed (Jang et al., 2017). Zhang and coworkers employed a more advanced strategy, where they used coaxial printing to pattern human umbilical vein endothelial cells (HUVECs) in a ink containing alginate, GelMA and the UV-curable photoinitiator Irgacure. The mixture was quickly crosslinked by CaCl_2_ from the outer sheath, and further gelled by exposure to UV light (for alginate and GelMA respectively) (Zhang Y. S. et al., 2016). HUVECs migrated to the outer border of the printed structures, forming 3D vessel-like structures over a period of 15 days, after which neonatal rat CMs were added to complete the tissue. Izadifar et al. explored varying the electrical properties of the generated tissues by incorporating alginate-coated carbon nanotubes (CNT) to methacrylated collagen. The system displayed as expected enhanced electrical properties, once more at the expense of becoming stiffer, though within acceptable values, and was able to support the growth of human coronary artery endothelial cells. No CMs were employed (Izadifar et al., 2018). Maiullari and coworkers applied a different version of extrusion printing termed microfluidic printing, to generate vascularised engineered tissues combining HUVECs and hiPSC-CMs (Maiullari et al., 2018). The bioartificial myocardium was fabricated with a bioink combining alginate for a rapid crosslinking, and polyethylene glycol (PEG) monoacrylate-fibrinogen, for UV-curing and cell adhesion. These tissues were tested *in vivo*, were the presence of vascular cells was able to promote integration with the recipient vascular system. Zhu et al. formulated a bioink with capacity to electrically influence neonatal rat CMs by incorporating gold nanorods in GelMA. Thick constructs were generated, although cell viability was below 80% in most cases. This is extremely relevant if we take into account that CMs are non-proliferative cells, so no re-growth is likely to occur (Zhu et al., 2017).

Freeform embedding of suspended hydrogels (FRESH) is one of most exciting 3D bioprinting methods (Hinton et al., 2015). Here, the biomaterial ink or bioink of choice is printed within the support of another hydrogel serving as a temporary support. Noor and co-workers developed a personalised hydrogel ink from human decellularised omental tissue, which could be printed into complex structures using FRESH. They used it in combination with hiPSC which they differentiated within the matrix into beating, perfused cardiac mini-tissues (Noor et al., 2019). Lastly, the Feinberg group used FRESH to accurately (20 μm resolution) 3D print different components of the human heart with collagen, including a model of the left ventricle on the scale of the neonatal organ, incorporating hESC-CMs (Lee et al., 2019). These engineered tissues displayed synchronised functional activity.

Finally, some the applications build tissues without the use of biomaterials. This includes the additive manufacturing with spheroids, as demonstrated by Arai et al., where they combined commercially available hPSC-CMs with HUVECs and dermal fibroblasts to generate the spheroids, organised in a tubular 3D structure with a bioprinter (Arai et al., 2018). However, the authors used a needle array to provide initial transient support for their structures, which in the end played the role of a scaffolding material. Recently, Ayan et al. reported a novel strategy, termed aspiration-assisted bioprinting, relying on the aspiration of 3D spheroids and their precise deposition in 3D. This could also be combined with FRESH, but no use of hiPSC-derived cardiac cells was reported (Ayan et al., 2020).

## Clinical Translation of hPSC-Based cTE Strategies

Survival post-cardiac ischemia has raised with advances in the cardiology and cardiovascular surgery areas, as well as through the development of new drugs in the last decades. This includes the application of reperfusion by percutaneous coronary intervention, anticoagulants and antithrombotics, amongst others. Though cTE has potential to address different cardiac conditions, its main activity has been directed towards the loss of viable myocardium by supplying new one, engineered in the lab.

In stark contrast to the short time elapsing between the first reports on regenerative medicine with adult stem cells and their first in-human application (reviewed in Banerjee et al., 2018) cTE with hPSCs has scarcely reached the clinical arena. Be it because both scientists and clinicians have learnt from past experiences or because translation presents additional complications, (Desgres and Menasché, 2019; Ghaemi et al., 2019; Ke and Murphy, 2019) clinical application is yet to become a reality. The technology faces challenges in the area of regulation, where reprogrammed cells under GMP-conditions are not widely derived, in logistics, with the size of engineered tissues in most instances requiring open chest surgery, and in economic terms. However, progress is being made. Tiburcy et al., created an engineered human myocardium with advanced structural and functional maturation by combining hPSC-derived cells with collagen type I (Tiburcy et al., 2017). Their work, standing on the shoulders of decade-long progress, (Zimmermann et al., 2002, 2006) demonstrated the building of human-scale relevant tissues (3.5 × 3.4 cm, containing 40 million CMs) under defined conditions and their pre-clinical safety assessment in rats, though no large animal testing was performed. The group of Prof Bursac applied dynamic culture conditions to improve the maturation of a patch formed from hiPSC-derived cardiac cells in a fibrin matrix. Although they achieved human adult-like electrical properties (e.g., conduction velocity) and formed large grafts when transplanted into rodents, the use of matrigel in the forming mixture precludes further translation (Shadrin et al., 2017; Jackman et al., 2018). In addition, myocytes were mostly randomly arrayed, which added to the low thickness of the grafts precludes any efficient and substantial force generation, and therefore makes providing contractile support to diseased hearts difficult.

Menasche et al. performed the first clinical case of an hPSC-based patch in 2015, transplanting a 20 cm^2^ fibrin-based tissue. This patch contained 4 million hESC-derived Isl^+^ SSEA1^+^ CPCs and was implanted epicardially in a 68-year-old patient with advanced heart failure. Concomitant to the implantation of the patch, a coronary artery bypass was also performed. After 3 months, the patient had better symptoms, and there was improved evidence in the echocardiogram too (form akinetic to moderately hypokinetic). Moreover, no adverse effect was observed (Menasché et al., 2015). This was part of the first clinical trial carried out with six patients suffering from advanced IHD (NCT02057900), called ESCORT. The aim of the trial was to demonstrate the safety of the patch at 1 year. All patients improved symptomatically, with four showing an increased systolic motion. One died early in the post-operative period from treatment-unrelated comorbidities and another after in 22 months due to heart failure (Menasché et al., 2018).

Japan has made huge efforts to spearhead research in the hiPSC field, and has one of the most streamlined regulatory frameworks (see Cyranoski, 2019 for a comment). It has recently started the first clinical trial of hiPSC-CM transplant in ischemic heart dysfunction. The study will deliver 100 million hiPSC-CMs in the form of a cell sheet of 4 × 5 × 0.1 cm (Kawamura et al., 2017). The aim of the study is to evaluate efficacy and safety of the treatment in a total of 10 patients (Cyranoski, 2018).

Overall, clinical applications of hPSC-based cardiac engineered tissues, though scarce, are starting to emerge. However, the scientific and logistic/economic concerns mentioned above need addressing further before any of these technologies reach the clinical arena.

## Conclusion and Outlook

The capacity to fabricate human myocardium in the laboratory is no longer restricted to science fiction. Recent breakthroughs such as cell reprogramming and 3D (bio)printing have shifted the question from ‘Can we do it?’ to ‘How do we want to do it?’. However, lessons from its predecessor in the area of regenerative medicine should teach us the value of caution. Crucial questions remain, some of which include the following:

-*What are the most suitable components for building an engineered myocardium?* Of course, this absolutely depends on the application/pathology we are targeting. If the objective is to reconstruct the post-disease myocardium, especially in cases when muscular mass has been loss (as IHD), this will require new CMs, alongside new vessels and support cells. Although not supplying new CMs might still be beneficial, it will not regenerate the tissue to pre-disease levels. So far, only hPSCs can produce the required cells in sufficiently large amounts, and whether it is better to use fully differentiated cells or progenitors is still an open question. Regarding the ECM-mimics, the current state can seem confusing. All reported (bio)material formulations seem to provide a benefit (Table 2). It is plausible that the most important factor is for the material to recapitulate the mechanical environment of cardiac tissue long enough for delivered cells to build their own structure. Also, although collagen type I has repeatedly been positioned as being the main component of the cardiac ECM, this does not account for all its mechanical capacities, nor it is as abundant as the levels supplemented in engineered tissues. Probably, cost and translation capacity will have much to say here. Lastly, regarding the fabrication mode, though 3D bioprinting has captivated both scientists and the wider public, its actual ability to deliver tissues able to withstand the mechanical forces in play in the human myocardium is far from optimal in most cases. Fibre reinforcement might be a solution to this, but remains to be fully tested in a human scale. In addition, recent technical developments such as 4D printing or cyborganics will certainly widen the scope of possibilities at hand (Tamay et al., 2019; Orive et al., 2020).

– *How is translation going to happen?* The hurdles are significant, (Desgres and Menasché, 2019; Ghaemi et al., 2019) not only due to the use of genetically modified cells (hiPSCs), exogenous materials and complex equipment, but also because in order to attain sufficient functional capacity, it is likely that some lengthy period of *in vitro* electromechanical maturation must be implemented. Also, no standard equipment currently exists to apply this to a human-scale tissue. In this area, it is crucial that biomedical researchers work alongside lawmakers. Cell therapy mostly relied on an autologous application of a non-cultured material, which smoothened its approval. Tissue engineering applications based on hiPSC products are highly promising but are impeded by stringent regulations. Japan has spearheaded hiPSC translation as a national flagship (Azuma and Yamanaka, 2016). Although some consider this too loose, it is clear that unless scientists provide a clear direction and get themselves involved in depth in the regulatory process, advances will be limited.

– *Will costs be sensible?* hiPSC culture and differentiation are expensive. Additive manufacturing is expensive. GMP-grade materials are expensive. hiPSC line derivation costs between €4000-8000 depending on the provider, multi-nozzle 3D bioprinters are usually budgeted above €30000, and some medical-grade materials as human collagen type I are prohibitive (> €200 per 100 μg). Highly trained personnel costs need to be added on top of this, as well as the cost of hiPSC expansion and differentiation. The cost of a heart transplant in the United States is above $150000 (including 120-day medical care), with Left Ventricular Assist Devices (LVADs), employed for patients with end stage heart failure as a bridge to transplant (and now trialled as destination therapy) are estimated to cost even more (see Miller et al., 2013 for a concise review). If economic issues are not addressed from day one, we run the risk of ending up with a highly effective therapy only that very few people can pay for. Large-scale production of hiPSC-derived cells has been demonstrated and will no doubt help decrease costs (Abecasis et al., 2017; Halloin et al., 2019; Buikema et al., 2020). Strategies to induce hiPSC-CM division would significantly improve the economics.

– *Will the new therapy be safe?* hPSCs have been marred by concerns related to persistence of pluripotent cells after differentiation and the subsequent risk of teratoma formation. But also, the new tissue must conform to the host’s electrical activity. Again, lessons from Cell Therapy call for caution (Menasché et al., 2008). Advanced genomics and other analysis tools not available 10 years ago will significantly enhance the safety analysis and help researchers and clinicians along the way (Wang Z. et al., 2020).

– *Are the aims achievable?* The objective of cTE is to generate new human myocardium whose properties and functionality are similar to its natural counterpart, but that might not be achievable within a sensible timeframe. Human cardiac maturation advances significantly after birth but continues throughout the first decades of life. Current advanced methods for maturation, like those based on electromechanical stimulation, do not achieve anything even close to adult properties. However, it is plausible that only a minimal maturity is needed in order to avoid lethal arrhythmias, as hPSC-CMs are reported to undergo maturation after transplantation in the heart (Kadota et al., 2017).

– *What is the state of non-therapeutic applications?* Though the aim of cTE within regenerative medicine is directed towards treatment, bioartificial human myocardium is becoming increasingly relevant with respect to other applications such as disease modelling and drug testing (Vunjak-Novakovic et al., 2014; Sharma et al., 2020), where the scale of the fabricated tissues is not an issue. In drug testing, the CiPA initiative has already pointed to the relevance of hiPSC-CMs, (Millard et al., 2018) especially given cardiac side effects are one of the main reasons for drug withdrawal, and there is no primary source of human CMs available (Laverty et al., 2011). Current efforts have already proven the capacity of cTE-based systems to better recapitulate physiological human drug response [reviewed in Kadota et al. (2017) and Millard et al. (2018), or to generate chamber-specific microtissues (Zhao et al., 2019)]. Translation towards industrial and clinical testing is envisioned to be more straightforward and immediate.

As can be seen, the challenges ahead are not minor. However, humankind has never been closer to achieving success in tissue fabrication. Whole functional heart generation in the laboratory, though frequently dreamt of, is still not achievable, as severe physiological and technical roadblocks remain in place. Challenges include ensuring adequate vascularisation, electromechanical coupling over long distances, atrioventricular delay, autonomous nervous system innervation or designing materials both biocompatible but capable of supporting the strong mechanics of a whole heart, amongst others. At this extraordinary moment, it is crucial for scientists, clinicians, and lay people to stay focused and deliver.

## Author Contributions

PM, MF-I, SM, and MP revised the literature, wrote the initial draft of the manuscript and figures. DP and JG revised the text and generated the tables. CS, GO, and FP supervised the development and revised the text. MM wrote the final revised version. All authors contributed to the article and approved the submitted version.

## Conflict of Interest

The authors declare that the research was conducted in the absence of any commercial or financial relationships that could be construed as a potential conflict of interest.

## References

[B1] Abecasis B. Aguiar T. Arnault É Costa R. Gomes-Alves P. Aspegren A. (2017). Expansion of 3D human induced pluripotent stem cell aggregates in bioreactors: bioprocess intensification and scaling-up approaches. *J. Biotechnol.* 246 81–93. 10.1016/j.jbiotec.2017.01.004 28131858

[B2] Adler C. P. Costabel U. (1975). Cell number in human heart in atrophy, hypertrophy, and under the influence of cytostatics. *Recent Adv. Stud. Card. Struct. Metab.* 6 343–355. 128080

[B3] Akbari M. Tamayol A. Bagherifard S. Serex L. Mostafalu P. Faramarzi N. (2016). Textile technologies and tissue engineering: a path toward organ weaving. *Adv. Healthc. Mater.* 5 751–766. 10.1002/adhm.201500517 26924450 PMC4910159

[B4] Amdursky N. Mazo M. M. Thomas M. R. Humphrey E. J. Puetzer J. L. St-Pierre J. P. (2018). Elastic serum-albumin based hydrogels: mechanism of formation and application in cardiac tissue engineering. *J. Mater. Chem. B* 6 5604–5612. 10.1039/c8tb01014e 30283632 PMC6166857

[B5] Andreu I. Luque T. Sancho A. Pelacho B. Iglesias-García O. Melo E. (2014). Heterogeneous micromechanical properties of the extracellular matrix in healthy and infarcted hearts. *Acta Biomater.* 10 3235–3242. 10.1016/j.actbio.2014.03.034 24717359

[B6] Anversa P. Loud A. V. Giacomelli F. Wiener J. (1978). Absolute morphometric study of myocardial hypertrophy in experimental hypertension. II. Ultrastructure of myocytes and interstitium. *Lab. Investig.* 38 597–609. 147963

[B7] Arai K. Murata D. Verissimo A. R. Mukae Y. Itoh M. Nakamura A. (2018). Fabrication of scaffold-free tubular cardiac constructs using a Bio-3D printer. *PLoS ONE* 13:e0209162. 10.1371/journal.pone.0209162 30557409 PMC6296519

[B8] Araña M. Gavira J. J. Peña E. González A. Abizanda G. Cilla M. (2014). Epicardial delivery of collagen patches with adipose-derived stem cells in rat and minipig models of chronic myocardial infarction. *Biomaterials* 35 143–151. 10.1016/j.biomaterials.2013.09.083 24119456

[B9] Ayan B. Heo D. N. Zhang Z. Dey M. Povilianskas A. Drapaca C. (2020). Aspiration-assisted bioprinting for precise positioning of biologics. *Sci. Adv.* 6:eaaw5111. 10.1126/sciadv.aaw5111 32181332 PMC7060055

[B10] Azuma K. Yamanaka S. (2016). Recent policies that support clinical application of induced pluripotent stem cell-based regenerative therapies. *Regen. Ther.* 4 36–47. 10.1016/j.reth.2016.01.009 31245486 PMC6581825

[B11] Banerjee I. Fuseler J. W. Price R. L. Borg T. K. Baudino T. A. (2007). Determination of cell types and numbers during cardiac development in the neonatal and adult rat and mouse. *Am. J. Physiol. – Hear Circ. Physiol.* 293 1883–1891 10.1152/ajpheart.00514.2007 17604329

[B12] Banerjee M. N. Bolli R. Hare J. M. (2018). Clinical studies of cell therapy in cardiovascular medicine recent developments and future directions. *Circ. Res.* 123 266–287. 10.1161/circresaha.118.311217 29976692 PMC8742222

[B13] Bargehr J. Ong L. P. Colzani M. Davaapil H. Hofsteen P. Bhandari S. (2019). Epicardial cells derived from human embryonic stem cells augment cardiomyocyte-driven heart regeneration. *Nat. Biotechnol.* 37 895–906. 10.1038/s41587-019-0197-9 31375810 PMC6824587

[B14] Barkauskas C. E. Noble P. W. Brigid L. M. Barkauskas C. E. Cronce M. J. Rackley C. R. (2013). Type 2 alveolar cells are stem cells in adult lung. *J. Clin. Invest.* 123 3025–3036. 23921127 10.1172/JCI68782 PMC3696553

[B15] Barreiro D. L. Martín-Moldes Z. Yeo J. Shen S. Hawker M. J. Martin-Martinez F. J. (2019). Conductive silk-based composites using biobased carbon materials. *Adv. Mater.* 31:e1904720. 10.1002/adma.201904720 PMC6824953 31532880

[B16] Barron J. Wu P. Ladouceur H. D. Ringeisen B. R. (2004). Biological laser printing: a novel technique for creating heterogeneous 3-dimensional cell patterns. *Biomed. Microdevices* 6 139–147. 10.1023/b:bmmd.0000031751.67267.9f 15320636

[B17] Bas O. D’Angella D. Baldwin J. G. Castro N. J. Wunner F. M. Saidy N. T. (2017). An integrated design, material, and fabrication platform for engineering biomechanically and biologically functional soft tissues. *ACS Appl. Mater. Interfaces.* 9 29430–29437. 10.1021/acsami.7b08617 28816441

[B18] Bas O. De-Juan-Pardo E. M. Chhaya M. P. Wunner F. M. Jeon J. E. Klein T. J. (2015). Enhancing structural integrity of hydrogels by using highly organised melt electrospun fibre constructs. *Eur. Polym. J.* 72 451–463. 10.1016/j.eurpolymj.2015.07.034

[B19] Baudino T. A. Carver W. Giles W. Borg T. K. (2006). Cardiac fibroblasts: friend or foe? *Am. J. Physiol. – Hear Circ. Physiol.* 291 H1015–H1026. 10.1152/ajpheart.00023.2006 16617141

[B20] Bedada F. B. Chan S. S. K. Metzger S. K. Zhang L. Zhang J. Garry D. J. (2014). Acquisition of a quantitative, stoichiometrically conserved ratiometric marker of maturation status in stem cell-derived cardiac myocytes. *Stem Cell Rep.* 3 594–605. 10.1016/j.stemcr.2014.07.012 25358788 PMC4223713

[B21] Behfar A. Crespo-Diaz R. Terzic A. Gersh B. J. (2014). Cell therapy for cardiac repair-lessons from clinical trials. *Nat. Rev. Cardiol.* 11 232–246. 10.1038/nrcardio.2014.9 24594893

[B22] Beltrami A. P. Barlucchi L. Torella D. Baker M. Limana F. Chimenti S. (2003). Adult cardiac stem cells are multipotent and support myocardial regeneration. *Cell* 114 763–776. 10.1016/s0092-8674(03)00687-1 14505575

[B23] Belviso I. Romano V. Sacco A. M. Ricci G. Massai D. Cammarota M. (2020). Decellularized human dermal matrix as a biological scaffold for cardiac repair and regeneration. *Front. Bioeng. Biotechnol.* 8:229. 10.3389/fbioe.2020.00229 32266249 PMC7099865

[B24] Bergmann O. Zdunek S. Felker A. Salehpour M. Alkass K. Bernard S. (2015). Dynamics of cell generation and turnover in the human heart. *Cell* 161 1566–1575. 10.1016/j.cell.2015.05.026 26073943

[B25] Bertlein S. Brown G. Lim K. S. Jungst T. Boeck T. Blunk T. (2017). Thiol-ene clickable gelatin: a platform bioink for multiple 3D biofabrication technologies. *Adv. Mater.* 29:1703404. 10.1002/adma.201703404 29044686

[B26] Bhana B. Iyer R. K. Chen W. L. K. Zhao R. Sider K. L. Likhitpanichkul M. (2010). Influence of substrate stiffness on the phenotype of heart cells. *Biotechnol. Bioeng.* 105 1148–1160. 20014437 10.1002/bit.22647

[B27] Birket M. J. Ribeiro M. C. Verkerk A. O. Ward D. Leitoguinho A. R. Den Hartogh S. C. (2015). Expansion and patterning of cardiovascular progenitors derived from human pluripotent stem cells. *Nat. Biotechnol.* 33 970–979. 10.1038/nbt.3271 26192318

[B28] Blin G. Nury D. Stefanovic S. Neri T. Guillevic O. Brinon B. (2010). A purified population of multipotent cardiovascular progenitors derived from primate pluripotent stem cells engrafts in postmyocardial infarcted nonhuman primates. *J. Clin. Invest.* 120 1125–1139. 10.1172/jci40120 20335662 PMC2846046

[B29] Bondue A. Lapouge G. Paulissen C. Semeraro C. Iacovino M. Kyba M. (2008). Mesp1 acts as a master regulator of multipotent cardiovascular progenitor specification. *Cell Stem Cell* 3 69–84. 10.1016/j.stem.2008.06.009 18593560

[B30] Brand T. (2003). Heart development: molecular insights into cardiac specification and early morphogenesis. *Dev. Biol.* 258 1–19. 10.1016/s0012-1606(03)00112-x 12781678

[B31] Bressan M. Liu G. Mikawa T. (2013). Early mesodermal cues assign avian cardiac pacemaker fate potential in a tertiary heart field. *Science(80-)* 340 744–748. 10.1126/science.1232877 23519212 PMC3651765

[B32] Brown T. D. Dalton P. D. Hutmacher D. W. (2011). Direct writing by way of melt electrospinning. *Adv. Mater.* 23 5651–5657. 10.1002/adma.201103482 22095922

[B33] Buckberg G. Nanda N. Nguyen C. Kocica M. (2018). What is the heart? Anatomy, function, pathophysiology, and misconceptions. *J. Cardiovasc Dev. Dis.* 5:33. 10.3390/jcdd5020033 29867011 PMC6023278

[B34] Buckberg G. D. (2002). Basic science review: the helix and the heart. *J. Thorac. Cardiovasc. Surg.* 124 863–883. 10.1067/mtc.2002.122439 12407367

[B35] Buckberg G. D. Hoffman J. I. E. Cecil Coghlan H. Nanda N. C. (2015a). Ventricular structure-function relations in health and disease: part I. The normal heart. *Eur. J. Cardio-thoracic Surg.* 47 587–601. 10.1093/ejcts/ezu278 25086103

[B36] Buckberg G. D. Hoffman J. I. E. Coghlan H. C. Nanda N. C. (2015b). Ventricular structure-function relations in health and disease: part II. Clinical considerations. *Eur. J. Cardio-thoracic. Surg.* 47 778–787. 10.1093/ejcts/ezu279 25082144

[B37] Buckingham M. Meilhac S. Zaffran S. (2005). Building the mammalian heart from two sources of myocardial cells. *Nat. Rev. Genet.* 6 826–835. 10.1038/nrg1710 16304598

[B38] Buikema J. W. Lee S. Goodyer W. R. Maas R. G. Chirikian O. Li G. (2020). Wnt activation and reduced cell-cell contact synergistically induce massive expansion of functional human iPSC-derived cardiomyocytes. *Cell Stem Cell* 27 50.e5–63.e5. 32619518 10.1016/j.stem.2020.06.001 PMC7334437

[B39] Burnett W. Finnigan-Bunick A. Yoon K. Rosenbloom J. (1982). Analysis of elastin gene expression in the developing chick aorta using cloned elastin cDNA. *J. Biol. Chem.* 257 1569–1572. 6895753

[B40] Burridge P. W. Anderson D. Priddle H. Barbadillo Muñoz M. D. Chamberlain S. Allegrucci C. (2007). Improved human embryonic stem cell embryoid body homogeneity and cardiomyocyte differentiation from a novel V-96 plate aggregation system highlights interline variability. *Stem Cells* 25 929–938. 10.1634/stemcells.2006-0598 17185609

[B41] Burridge P. W. Matsa E. Shukla P. Lin Z. C. Churko J. M. Ebert A. D. (2014). Chemically defned generation of human cardiomyocytes. *Nat. Methods* 11 855–860. 10.1038/nmeth.2999 24930130 PMC4169698

[B42] Burridge P. W. Sharma A. Wu J. C. (2015). Genetic and epigenetic regulation of human cardiac reprogramming and differentiation in regenerative medicine. *Annu. Rev. Genet.* 49 461–484. 10.1146/annurev-genet-112414-054911 26631515 PMC4962619

[B43] Camelliti P. Borg T. K. Kohl P. (2005). Structural and functional characterisation of cardiac fibroblasts. *Cardiovasc. Res.* 65 40–51. 10.1016/j.cardiores.2004.08.020 15621032

[B44] Camelliti P. Green C. R. LeGrice I. Kohl P. (2004). Fibroblast network in rabbit sinoatrial node: structural and functional identification of homogeneous and heterogeneous cell coupling. *Circ. Res.* 94 828–835. 10.1161/01.res.0000122382.19400.14 14976125

[B45] Castilho M. Feyen D. Flandes-Iparraguirre M. Hochleitner G. Groll J. Doevendans P. A. F. (2017). Melt electrospinning writing of poly-hydroxymethylglycolide-co-ε-caprolactone-based scaffolds for cardiac tissue engineering. *Adv. Healthc. Mater.* 6:1700311. 10.1002/adhm.201700311 PMC7116102 28699224

[B46] Castilho M. van Mil A. Maher M. Metz C. Hochleitner G. Groll J. (2018). Melt electrowriting allows tailored microstructural and mechanical design of scaffolds to advance functional human myocardial tissue formation. *Adv. Funct. Mater.* 28:1803151 10.1002/adfm.201803151

[B47] Chan S. S. K. Shi X. Toyama A. Arpke R. W. Dandapat A. Iacovino M. (2013). Mesp1 patterns mesoderm into cardiac, hematopoietic, or skeletal myogenic progenitors in a context-dependent manner. *Cell Stem Cell* 12 587–601. 10.1016/j.stem.2013.03.004 23642367 PMC3646300

[B48] Chan Y. C. Ting S. Lee Y. K. Ng K. M. Zhang J. Chen Z. (2013). Electrical stimulation promotes maturation of cardiomyocytes derived from human embryonic stem cells. *J. Cardiovasc. Transl. Res.* 6 989–999. 10.1007/s12265-013-9510-z 24081385

[B49] Chen Q. Z. Bismarck A. Hansen U. Junaid S. Tran M. Q. Harding S. E. (2008). Characterisation of a soft elastomer poly(glycerol sebacate) designed to match the mechanical properties of myocardial tissue. *Biomaterials* 29 47–57. 10.1016/j.biomaterials.2007.09.010 17915309

[B50] Christoffels V. M. Burch J. B. E. Moorman A. F. M. (2004). Architectural plan for the heart: early patterning and delineation of the chambers and the nodes. *Trends Cardiovasc. Med.* 14 301–307. 10.1016/j.tcm.2004.09.002 15596106

[B51] Chung C. Y. Bien H. Entcheva E. (2007). The role of cardiac tissue alignment in modulating electrical function. *J. Cardiovasc. Electrophysiol.* 18 1323–1329. 10.1111/j.1540-8167.2007.00959.x 17916158

[B52] Crowder S. W. Leonardo V. Whittaker T. Papathanasiou P. Stevens M. M. (2016). Material cues as potent regulators of epigenetics and stem cell function. *Cell Stem Cell* 18 39–52. 10.1016/j.stem.2015.12.012 26748755 PMC5409508

[B53] Cui X. Soliman B. G. Alcala-Orozco C. R. Li J. Vis M. A. M. Santos M. (2020). Rapid photocrosslinking of silk hydrogels with high cell density and enhanced shape fidelity. *Adv. Healthc. Mater.* 9:e1901667. 10.1002/adhm.201901667 31943911

[B54] Cyranoski D. (2018). “Reprogrammed” stem cells approved to mend human hearts for the first time. *Nature* 557 619–620. 10.1038/d41586-018-05278-8 29844563

[B55] Cyranoski D. (2019). The potent effects of Japan’s stem-cell policies. *Nature* 573 482–485. 10.1038/d41586-019-02847-3 31554988

[B56] Dattola E. Parrotta E. I. Scalise S. Perozziello G. Limongi T. Candeloro P. (2019). Development of 3D PVA scaffolds for cardiac tissue engineering and cell screening applications. *RSC Adv.* 9 4246–4257. 10.1039/c8ra08187e PMC9060459 35520194

[B57] Davidson J. M. Smith K. Shibahara S. Tolstoshev P. Crystal R. G. (1982). Regulation of elastin synthesis in developing sheep nuchal ligament by elastin mRNA levels. *J. Biol. Chem.* 257 747–754. 7054180

[B58] Desgres M. Menasché P. (2019). Clinical translation of pluripotent stem cell therapies: challenges and considerations. *Cell Stem Cell* 25 594–606. 10.1016/j.stem.2019.10.001 31703770

[B59] Doetsch F. Caille I. Lim D. A. Garcı J. M. Alvarez-buylla A. (1999). Subventricular zone astrocytes are neural stem cells in the adult mammalian brain. *Cell* 97 703–716. 10.1016/s0092-8674(00)80783-7 10380923

[B60] Domian I. J. Yu H. Mittal N. (2017). On materials for cardiac tissue engineering. *Adv. Healthc. Mater.* 6:1600768. 10.1002/adhm.201600768 27774763

[B61] Du D. T. M. Hellen N. Kane C. Terracciano C. M. N. (2015). Action potential morphology of human induced pluripotent stem cell-derived cardiomyocytes does not predict cardiac chamber specificity and is dependent on cell density. *Biophys. J.* 108 1–4. 10.1016/j.bpj.2014.11.008 25564842 PMC4286594

[B62] Duan B. Wang M. Zhou W. Y. Cheung W. L. Li Z. Y. Lu W. W. (2010). Three-dimensional nanocomposite scaffolds fabricated via selective laser sintering for bone tissue engineering. *Acta Biomater.* 6 4495–4505. 10.1016/j.actbio.2010.06.024 20601244

[B63] Dubick M. A. Rucker R. B. Cross C. E. Last J. A. (1981). Elastin metabolism in rodent lung. *BBA – Gen. Subj.* 672 303–306. 10.1016/0304-4165(81)90297-x 7213816

[B64] Eder A. Vollert I. Hansen A. Eschenhagen T. (2016). Human engineered heart tissue as a model system for drug testing. *Adv. Drug Deliv. Rev.* 96 214–224. 10.1016/j.addr.2015.05.010 26026976

[B65] Eghbali M. Czaja M. J. Zeydel M. Weiner F. R. Zern M. A. Seifter S. (1988). Collagen chain mRNAs in isolated heart cells from young and adult rats. *J. Mol. Cell. Cardiol.* 20 267–276. 10.1016/s0022-2828(88)80059-2 3398057

[B66] Eghbali M. Weber K. T. (1990). Collagen and the myocardium: fibrillar structure, biosynthesis and degradation in relation to hypertrophy and its regression. *Mol. Cell. Biochem.* 96 1–14. 2146489 10.1007/BF00228448

[B67] Elliott D. A. Braam S. R. Koutsis K. Ng E. S. Jenny R. Lagerqvist E. L. (2011). NKX2-5eGFP/w hESCs for isolation of human cardiac progenitors and cardiomyocytes. *Nat. Methods* 8 1037–1040. 10.1038/nmeth.1740 22020065

[B68] Engler A. J. Carag-Krieger C. Johnson C. P. Raab M. Tang H. Y. Speicher D. W. (2008). Embryonic cardiomyocytes beat best on a matrix with heart-like elasticity: scar-like rigidity inhibits beating. *J. Cell Sci.* 121 3794–3802. 10.1242/jcs.029678 18957515 PMC2740334

[B69] Eschenhagen T. Bolli R. Braun T. Field L. J. Fleischmann B. K. Frisén J. (2017). Cardiomyocyte regeneration: a consensus statement. *Circulation* 136 680–686. 10.1161/circulationaha.117.029343 28684531 PMC5557671

[B70] Eschenhagen T. Fink C. Remmers U. Scholz H. Wattchow J. Weil J. (1997). Three-dimensional reconstitution of embryonic cardiomyocytes in a collagen matrix: a new heart muscle model system. *FASEB J.* 11 683–694. 10.1096/fasebj.11.8.9240969 9240969

[B71] Fan D. Takawale A. Lee J. Kassiri Z. (2012). Cardiac fibroblasts, fibrosis and extracellular matrix remodeling in heart disease. *Fibrogenes Tissue Repair.* 5 1–13. 10.1186/1755-1536-5-15 PMC3464725 22943504

[B72] Feaster T. K. Cadar A. G. Wang L. Williams C. H. Chun Y. W. Hempel J. E. (2015). Matrigel mattress: a method for the generation of single contracting human-induced pluripotent stem cell-derived cardiomyocytes. *Circ Res.* 117 995–1000. 10.1161/circresaha.115.307580 26429802 PMC4670592

[B73] Feric N. T. Pallotta I. Singh R. Bogdanowicz D. R. Gustilo M. M. Chaudhary K. W. (2019). Engineered cardiac tissues generated in the biowire II: a platform for human-based drug discovery. *Toxicol. Sci.* 172 89–97. 10.1093/toxsci/kfz168 31385592 PMC6813749

[B74] Fink C. Ergün S. Kralisch D. Remmers U. Weil J. Eschenhagen T. (2000). Chronic stretch of engineered heart tissue induces hypertrophy and functional improvement. *FASEB J.* 14 669–679. 10.1096/fasebj.14.5.669 10744624

[B75] Fleischer S. Shapira A. Feiner R. Dvir T. (2017). Modular assembly of thick multifunctional cardiac patches. *Proc. Natl. Acad. Sci. U.S.A.* 114 1898–1903. 10.1073/pnas.1615728114 28167795 PMC5338434

[B76] Fleischer S. Shapira A. Regev O. Nseir N. Zussman E. Dvir T. (2014). Albumin fiber scaffolds for engineering functional cardiac tissues. *Biotechnol. Bioeng.* 111 1246–1257. 10.1002/bit.25185 24420414

[B77] Fomovsky G. M. Thomopoulos S. Holmes J. W. (2010). Contribution of extracellular matrix to the mechanical properties of the heart. *J. Mol. Cell Cardiol.* 48 490–496. 10.1016/j.yjmcc.2009.08.003 19686759 PMC2823835

[B78] Forrest L. Jackson D. (1971). Intermolecular cross-linking of collagen in human and guinea pig scar tissue. *Biochim. Biophys. Acta Protein Struct.* 229 681–689. 10.1016/0005-2795(71)90284-4 4929150

[B79] Frangogiannis N. G. (2019). The extracellular matrix in ischemic and nonischemic heart failure. *Circ Res.* 125 117–146. 10.1161/circresaha.119.311148 31219741 PMC6588179

[B80] Gaebel R. Ma N. Liu J. Guan J. Koch L. Klopsch C. (2011). Patterning human stem cells and endothelial cells with laser printing for cardiac regeneration. *Biomaterials* 32 9218–9230. 10.1016/j.biomaterials.2011.08.071 21911255

[B81] Ganji Y. Li Q. Quabius E. S. Böttner M. Selhuber-Unkel C. Kasra M. (2016). Cardiomyocyte behavior on biodegradable polyurethane/gold nanocomposite scaffolds under electrical stimulation. *Mater. Sci. Eng. C* 59 10–18. 10.1016/j.msec.2015.09.074 26652343

[B82] Garreta E. de Oñate L. Fernández-Santos M. E. Oria R. Tarantino C. Climent A. M. (2016). Myocardial commitment from human pluripotent stem cells: rapid production of human heart grafts. *Biomaterials* 1 64–78. 10.1016/j.biomaterials.2016.04.003 27179434

[B83] Gaudesius G. Miragoli M. Thomas S. P. Rohr S. (2003). Coupling of cardiac electrical activity over extended distances by fibroblasts of cardiac origin. *Circ Res.* 93 421–428. 10.1161/01.res.0000089258.40661.0c 12893743

[B84] Gauvin R. Chen Y. C. Lee J. W. Soman P. Zorlutuna P. Nichol J. W. (2012). Microfabrication of complex porous tissue engineering scaffolds using 3D projection stereolithography. *Biomaterials* 33 3824–3834. 10.1016/j.biomaterials.2012.01.048 22365811 PMC3766354

[B85] Ghaemi R. V. Siang L. C. Yadav V. G. (2019). Improving the rate of translation of tissue engineering products. *Adv. Healthc. Mater.* 8:e1900538. 10.1002/adhm.201900538 31386306

[B86] Giacomelli E. Meraviglia V. Campostrini G. Cochrane A. Cao X. van Helden R. W. J. (2020). Human-iPSC-derived cardiac stromal cells enhance maturation in 3D cardiac microtissues and reveal non-cardiomyocyte contributions to heart disease. *Cell Stem Cell* 26 862.e11–879.e11. 32459996 10.1016/j.stem.2020.05.004 PMC7284308

[B87] Giannopoulos A. A. Mitsouras D. Yoo S. J. Liu P. P. Chatzizisis Y. S. Rybicki F. J. (2016). Applications of 3D printing in cardiovascular diseases. *Nat. Rev. Cardiol.* 13 701–718. 27786234 10.1038/nrcardio.2016.170

[B88] Gilbert S. H. Benson A. P. Li P. Holden A. V. (2007). Regional localisation of left ventricular sheet structure: integration with current models of cardiac fibre, sheet and band structure. *Eur. J. Cardio-Thoracic. Surg.* 32 231–249. 10.1016/j.ejcts.2007.03.032 17462906

[B89] Godier-Furnémont A. F. G. Tiburcy M. Wagner E. Dewenter M. Lämmle S. El-Armouche A. (2015). Physiologic force-frequency response in engineered heart muscle by electromechanical stimulation. *Biomaterials* 1 82–91. 10.1016/j.biomaterials.2015.03.055 25985155 PMC4921199

[B90] Goldfracht I. Efraim Y. Shinnawi R. Kovalev E. Huber I. Gepstein A. (2019). Engineered heart tissue models from hiPSC-derived cardiomyocytes and cardiac ECM for disease modeling and drug testing applications. *Acta Biomater.* 92 145–159. 10.1016/j.actbio.2019.05.016 31075518

[B91] Günthel M. Barnett P. Christoffels V. M. (2018). Development, proliferation, and growth of the mammalian heart. *Mol. Ther.* 26 1599–1609. 10.1016/j.ymthe.2018.05.022 29929790 PMC6037201

[B92] Guo Y. Pu W. T. (2020). Cardiomyocyte maturation: new phase in development. *Circ. Res.* 126 1086–1106. 10.1161/circresaha.119.315862 32271675 PMC7199445

[B93] Gyöngyösi M. Wojakowski W. Navarese E. P. Moye L. (2016). Meta-analyses of human cell-based cardiac regeneration therapies: controversies in meta-analyses results on cardiac cell-based regenerative studies. *Circ. Res.* 118 1254–1263. 10.1161/circresaha.115.307347 27081108 PMC4834852

[B94] Halloin C. Coffee M. Manstein F. Zweigerdt R. (2019). Production of cardiomyocytes from human pluripotent stem cells by bioreactor technologies. *Methods Mol. Biol*. 1994 55–70. 10.1007/978-1-4939-9477-9_5 31124104

[B95] Han J. Wu Q. Xia Y. Wagner M. B. Xu C. (2016). Cell alignment induced by anisotropic electrospun fibrous scaffolds alone has limited effect on cardiomyocyte maturation. *Stem Cell Res.* 16 740–750. 10.1016/j.scr.2016.04.014 27131761 PMC4903921

[B96] Happe C. L. Engler A. J. (2016). Mechanical forces reshape differentiation cues that guide cardiomyogenesis. *Circ. Res.* 118 296–310. 10.1161/circresaha.115.305139 26838315 PMC4743530

[B97] Hasenfuss G. Mulieri L. A. Blanchard E. M. Holubarsch C. Leavitt B. J. Ittleman F. (1991). Energetics of isometric force development in control and volume-overload human myocardium, Comparison with animal species. *Circ Res.* 68 836–846. 10.1161/01.res.68.3.836 1742869

[B98] Hattori F. Chen H. Yamashita H. Tohyama S. Satoh Y.-S. Yuasa S. (2010). Nongenetic method for purifying stem cell-derived cardiomyocytes. *Nat. Methods* 7 61–66. 10.1038/nmeth.1403 19946277

[B99] Herget G. W. Neuburger M. Plagwitz R. Adler C. P. (1997). DNA content, ploidy level and number of nuclei in the human heart after myocardial infarction. *Cardiovasc. Res.* 36 45–51. 10.1016/s0008-6363(97)00140-5 9415271

[B100] Hernández D. Millard R. Sivakumaran P. Wong R. C. B. Crombie D. E. Hewitt A. W. (2016). Electrical stimulation promotes cardiac differentiation of human induced pluripotent stem cells. *Stem Cells Int.* 2016 1718041. 10.1155/2016/1718041 PMC4691644 26788064

[B101] Herron T. J. Da Rocha A. M. Campbell K. F. Ponce-Balbuena D. Willis B. C. Guerrero-Serna G. (2016). Extracellular matrix-mediated maturation of human pluripotent stem cell-derived cardiac monolayer structure and electrophysiological function. *Circ. Arrhythmia Electrophysiol.* 9 1–12. 10.1161/CIRCEP.113.003638 PMC4833010 27069088

[B102] Hildreth V. Webb S. Bradshaw L. Brown N. A. Anderson R. H. Henderson D. J. (2008). Cells migrating from the neural crest contribute to the innervation of the venous pole of the heart. *J. Anat.* 212 1–11. 18031480 10.1111/j.1469-7580.2007.00833.x PMC2423382

[B103] Hinton T. J. Jallerat Q. Palchesko R. N. Park J. H. Grodzicki M. S. Shue H. J. (2015). Three-dimensional printing of complex biological structures by freeform reversible embedding of suspended hydrogels. *Sci. Adv.* 1:e1500758. 10.1126/sciadv.1500758 26601312 PMC4646826

[B104] Hirt M. N. Boeddinghaus J. Mitchell A. Schaaf S. Börnchen C. Müller C. (2014). Functional improvement and maturation of rat and human engineered heart tissue by chronic electrical stimulation. *J. Mol. Cell Cardiol.* 74 151–161. 10.1016/j.yjmcc.2014.05.009 24852842

[B105] Hochleitner G. Fürsattel E. Giesa R. Groll J. Schmidt H.-W. Dalton P. D. (2018). Melt electrowriting of thermoplastic elastomers. *Macromol. Rapid Commun.* 39:e1800055. 10.1002/marc.201800055 29656556

[B106] Hodgkinson C. P. Bareja A. Gomez J. A. Dzau V. J. (2016). Emerging concepts in paracrine mechanisms in regenerative cardiovascular medicine and biology. *Circ Res.* 118 95–107. 10.1161/circresaha.115.305373 26837742 PMC4874329

[B107] Holland C. Numata K. Rnjak-Kovacina J. Seib F. P. (2019). The biomedical use of silk: past, present, future. *Adv. Healthc. Mater.* 8:e1800465. 10.1002/adhm.201800465 30238637

[B108] Holopainen T. Räsänen M. Anisimov A. Tuomainen T. Zheng W. Tvorogov D. (2015). Endothelial Bmx tyrosine kinase activity is essential for myocardial hypertrophy and remodeling. *Proc. Natl. Acad. Sci. U.S.A.* 112 13063–13068. 10.1073/pnas.1517810112 26430242 PMC4620883

[B109] Hu Q. Sun X. Z. Parmenter C. D. J. Fay M. W. Smith E. F. Rance G. A. (2017). Additive manufacture of complex 3D Au-containing nanocomposites by simultaneous two-photon polymerisation and photoreduction. *Sci. Rep.* 7 1–9. 29215026 10.1038/s41598-017-17391-1 PMC5719407

[B110] Hulsmans M. Clauss S. Xiao L. Aguirre A. D. King K. R. Hanley A. (2017). Macrophages facilitate electrical conduction in the heart. *Cell* 169 510.e20–522.e20. 28431249 10.1016/j.cell.2017.03.050 PMC5474950

[B111] Ivashchenko C. Y. Pipes G. C. Lozinskaya I. M. Lin Z. Xiaoping X. Needle S. (2013). Human-induced pluripotent stem cell-derived cardiomyocytes exhibit temporal changes in phenotype. *Am. J. Physiol. – Hear Circ. Physiol.* 305 H913–H922. 10.1152/ajpheart.00819.2012 23832699

[B112] Iyer R. K. Chiu L. L. Y. Radisic M. (2009). Microfabricated poly(ethylene glycol) templates enable rapid screening of triculture conditions for cardiac tissue engineering. *J. Biomed. Mater. Res. – Part A* 89 616–631. 10.1002/jbm.a.32014 18442120

[B113] Izadifar M. Chapman D. Babyn P. Chen X. Kelly M. E. (2018). UV-assisted 3D bioprinting of nanoreinforced hybrid cardiac patch for myocardial tissue engineering. *Tissue Eng. Part C Methods* 24 74–88. 10.1089/ten.tec.2017.0346 29050528

[B114] Jackman C. P. Carlson A. L. Bursac N. (2016). Dynamic culture yields engineered myocardium with near-adult functional output. *Biomaterials* 1 66–79. 10.1016/j.biomaterials.2016.09.024 27723557 PMC5074846

[B115] Jackman C. P. Ganapathi A. M. Asfour H. Qian Y. Allen B. W. Li Y. (2018). Engineered cardiac tissue patch maintains structural and electrical properties after epicardial implantation. *Biomaterials* 1 48–58. 10.1016/j.biomaterials.2018.01.002 29309993 PMC5801076

[B116] Jacot J. G. Martin J. C. Hunt D. L. (2010). Mechanobiology of cardiomyocyte development. *J. Biomech.* 43 93–98. 10.1016/j.jbiomech.2009.09.014 19819458 PMC2813357

[B117] Jacot J. G. McCulloch A. D. Omens J. H. (2008). Substrate stiffness affects the functional maturation of neonatal rat ventricular myocytes. *Biophys J.* 1 3479–3487. 10.1529/biophysj.107.124545 18586852 PMC2547444

[B118] James D. Nam H. Seandel M. Nolan D. Janovitz T. Tomishima M. (2010). Expansion and maintenance of human embryonic stem cell–derived endothelial cells by TGFb inhibition is Id1 dependent. *Nat. Biotechnol.* 28 161–167. 20081865 10.1038/nbt.1605 PMC2931334

[B119] Jang J. Park H. J. Kim S. W. Kim H. Park J. Y. Na S. J. (2017). 3D printed complex tissue construct using stem cell-laden decellularized extracellular matrix bioinks for cardiac repair. *Biomaterials* 112 264–274. 10.1016/j.biomaterials.2016.10.026 27770630

[B120] Janik H. Marzec M. (2015). A review: fabrication of porous polyurethane scaffolds. *Mater. Sci. Eng. C* 48 586–591. 10.1016/j.msec.2014.12.037 25579961

[B121] Jiang Y. Park P. Hong S. M. Ban K. (2018). Maturation of cardiomyocytes derived from human pluripotent stem cells: current strategies and limitations. *Mol. Cells* 41 613–621. 29890820 10.14348/molcells.2018.0143 PMC6078855

[B122] Joanne P. Kitsara M. Boitard S. E. Naemetalla H. Vanneaux V. Pernot M. (2016). Nanofibrous clinical-grade collagen scaffolds seeded with human cardiomyocytes induces cardiac remodeling in dilated cardiomyopathy. *Biomaterials* 80 157–168. 10.1016/j.biomaterials.2015.11.035 26708641

[B123] Jong F. D. Virágh S. Moorman A. F. M. (1997). Cardiac development: a morphologically integrated molecular approach. *Compar. Toxicol.* 7 131–146. 10.1017/s1047951100009379

[B124] Jungst T. Smolan W. Schacht K. Scheibel T. Groll J. (2016). Strategies and molecular design criteria for 3D printable hydrogels. *Chem. Rev.* 116 1496–1539. 10.1021/acs.chemrev.5b00303 26492834

[B125] Kadota S. Minami I. Morone N. Heuser J. E. Agladze K. Nakatsuji N. (2013). Development of a reentrant arrhythmia model in human pluripotent stem cell-derived cardiac cell sheets. *Eur. Heart J.* 34 1147–1156. 10.1093/eurheartj/ehs418 23201623

[B126] Kadota S. Pabon L. Reinecke H. Murry C. E. (2017). In vivo maturation of human induced pluripotent stem cell-derived cardiomyocytes in neonatal and adult rat hearts. *Stem Cell Rep.* 8 278–289. 10.1016/j.stemcr.2016.10.009 28065644 PMC5311430

[B127] Kapnisi M. Mansfield C. Marijon C. Guex A. G. Perbellini F. Bardi I. (2018). Auxetic cardiac patches with tunable mechanical and conductive properties toward treating myocardial infarction. *Adv. Funct Mater.* 28:1800618. 10.1002/adfm.201800618 29875619 PMC5985945

[B128] Karbassi E. Fenix A. Marchiano S. Muraoka N. Nakamura K. Yang X. (2020). Cardiomyocyte maturation: advances in knowledge and implications for regenerative medicine. *Nat. Rev. Cardiol.* 17 341–359. 10.1038/s41569-019-0331-x 32015528 PMC7239749

[B129] Kattman S. J. Huber T. L. Keller G. M. M. (2006). Multipotent Flk-1+ cardiovascular progenitor cells give rise to the cardiomyocyte, endothelial, and vascular smooth muscle lineages. *Dev. Cell* 11 723–732. 10.1016/j.devcel.2006.10.002 17084363

[B130] Kattman S. J. Witty A. D. Gagliardi M. Dubois N. C. Niapour M. Hotta A. (2011). Stage-specific optimization of activin/nodal and BMP signaling promotes cardiac differentiation of mouse and human pluripotent stem cell lines. *Cell Stem Cell* 8 228–240. 10.1016/j.stem.2010.12.008 21295278

[B131] Katz T. C. Singh M. K. Degenhardt K. Rivera-Feliciano J. Johnson R. L. Epstein J. A. (2012). Distinct compartments of the proepicardial organ give rise to coronary vascular endothelial cells. *Dev. Cell* 22 639–650. 10.1016/j.devcel.2012.01.012 22421048 PMC3306604

[B132] Kawamura M. Miyagawa S. Fukushima S. Saito A. Miki K. Funakoshi S. (2017). Enhanced therapeutic effects of human iPS cell derived-cardiomyocyte by combined cell-sheets with omental flap technique in porcine ischemic cardiomyopathy model. *Sci. Rep.* 7:8824. 10.1038/s41598-017-08869-z PMC5562896 28821761

[B133] Ke D. Murphy S. V. (2019). Current challenges of bioprinted tissues towards clinical translation. *Tissue Eng. Part B Rev.* 25 1–13. 10.1089/ten.teb.2018.0132 30129878

[B134] Kehat I. Kenyagin-Karsenti D. Snir M. Segev H. Amit M. Gepstein A. (2001). Human embryonic stem cells can differentiate into myocytes with structural and functional properties of cardiomyocytes. *J. Clin. Invest.* 108 407–414. 10.1172/jci200112131 11489934 PMC209357

[B135] Keyte A. L. Alonzo-Johnsen M. Hutson M. R. (2014). Evolutionary and developmental origins of the cardiac neural crest: building a divided outflow tract. *Birth Defects Res. Part C – Embryo Today Rev.* 102 309–323. 10.1002/bdrc.21076 25227322 PMC4288758

[B136] Khan M. Xu Y. Hua S. Johnson J. Belevych A. Janssen P. M. L. (2015). Evaluation of changes in morphology and function of human induced pluripotent stem cell derived cardiomyocytes (hiPSC-CMs) cultured on an aligned-nanofiber cardiac patch. *PLoS ONE* 10:e0126338. 10.1371/journal.pone.0126338 25993466 PMC4437999

[B137] Kharaziha M. Nikkhah M. Shin S. R. Annabi N. Masoumi N. Gaharwar A. K. (2013). PGS:Gelatin nanofibrous scaffolds with tunable mechanical and structural properties for engineering cardiac tissues. *Biomaterials* 34 6355–6366. 10.1016/j.biomaterials.2013.04.045 23747008 PMC3685203

[B138] Kharaziha M. Shin S. R. Nikkhah M. Topkaya S. N. Masoumi N. Annabi N. (2014). Tough and flexible CNT-polymeric hybrid scaffolds for engineering cardiac constructs. *Biomaterials* 35 7346–7354. 10.1016/j.biomaterials.2014.05.014 24927679 PMC4114042

[B139] Kielty C. M. Shuttleworth M. J. Sherratt Adrian C. (2002). Elastic fibres. *J. Cell Sci.* 115 2817–2828. 12082143 10.1242/jcs.115.14.2817

[B140] Kim J. Wende A. R. Sena S. Theobald H. A. Soto J. Sloan C. (2008). Insulin-like growth factor I receptor signaling is required for exercise-induced cardiac hypertrophy. *Mol. Endocrinol.* 22 2532–2543. 10.1210/me.2008-0265 PMC2582541 18801929

[B141] Kim T. H. An D. B. Oh S. H. Kang M. K. Song H. H. Lee J. H. (2015). Creating stiffness gradient polyvinyl alcohol hydrogel using a simple gradual freezing-thawing method to investigate stem cell differentiation behaviors. *Biomaterials* 40 51–60. 10.1016/j.biomaterials.2014.11.017 25467820

[B142] Kitsara M. Agbulut O. Kontziampasis D. Chen Y. Menasché P. (2017). Fibers for hearts: a critical review on electrospinning for cardiac tissue engineering. *Acta Biomater.* 48 20–40. 10.1016/j.actbio.2016.11.014 27826001

[B143] Klabunde R. (2012). “Cellular structure and function,” in *Cardiovascular Physiology Concepts*, 2nd Edn, ed. Taylor C. (Philadelphia, PA: Lippincott Williams & Wilkins), 41–58.

[B144] Koch L. Kuhn S. Sorg H. Gruene M. Schlie S. Gaebel R. (2010). Laser printing of skin cells and human stem cells. *Tissue Eng. Part C Methods* 16 847–854. 10.1089/ten.tec.2009.0397 19883209

[B145] Kohl P. (2004). Cardiac cellular heterogeneity and remodelling. *Cardiovasc. Res.* 64 195–197. 10.1016/j.cardiores.2004.08.011 15485677

[B146] Koshy S. T. Desai R. M. Joly P. Li J. Bagrodia R. K. Lewin S. A. (2016). Click-crosslinked injectable gelatin hydrogels. *Adv. Healthc. Mater.* 5 541–547. 10.1002/adhm.201500757 26806652 PMC4849477

[B147] Krieg M. Arboleda-Estudillo Y. Puech P. H. Käfer J. Graner F. Müller D. J. (2008). Tensile forces govern germ-layer organization in zebrafish. *Nat. Cell Biol.* 10 429–436. 10.1038/ncb1705 18364700

[B148] Kumar A. Placone J. K. Engler A. J. (2017). Understanding the extracellular forces that determine cell fate and maintenance. *Development* 144 4261–4270. 10.1242/dev.158469 29183939 PMC5769638

[B149] Laflamme M. A. Chen K. Y. Naumova A. V. Muskheli V. Fugate J. A. Dupras S. K. (2007). Cardiomyocytes derived from human embryonic stem cells in pro-survival factors enhance function of infarcted rat hearts. *Nat. Biotechnol.* 25 1015–1024. 10.1038/nbt1327 17721512

[B150] Lavergne M. Derkaoui M. Delmau C. Letourneur D. Uzan G. Le Visage C. (2012). Porous polysaccharide-based scaffolds for human endothelial progenitor cells. *Macromol. Biosci.* 12 901–910. 10.1002/mabi.201100431 22696505

[B151] Laverty H. G. Benson C. Cartwright E. J. Cross M. J. Garland C. Hammond T. (2011). How can we improve our understanding of cardiovascular safety liabilities to develop safer medicines? *Br. J. Pharmacol.* 163 675–693. 10.1111/j.1476-5381.2011.01255.x 21306581 PMC3111672

[B152] Lee A. Hudson A. R. Shiwarski D. J. Tashman J. W. Hinton T. J. Yerneni S. (2019). 3D bioprinting of collagen to rebuild components of the human heart. *Science (80-)* 365 482–487. 10.1126/science.aav9051 31371612

[B153] LeGrice I. J. Takayama Y. Covell J. W. (1995). Transverse shear along myocardial cleavage planes provides a mechanism for normal systolic wall thickening. *Circ. Res.* 77 182–193. 10.1161/01.res.77.1.182 7788876

[B154] Leucker T. M. Jones S. P. (2014). Endothelial dysfunction as a nexus for endothelial cell-cardiomyocyte miscommunication. *Front. Physiol.* 5:328. 10.3389/fphys.2014.00328 25206341 PMC4144117

[B155] Levenberg S. Golub J. S. Amit M. Itskovitz-Eldor J. Langer R. (2002). Endothelial cells derived from human embryonic stem cells. *Proc. Natl. Acad. Sci. U.S.A.* 99 4391–4396. 11917100 10.1073/pnas.032074999 PMC123658

[B156] Lian X. Hsiao C. Wilson G. Zhu K. Hazeltine L. B. Azarin S. M. (2012). Robust cardiomyocyte differentiation from human pluripotent stem cells via temporal modulation of canonical Wnt signaling. *Proc. Natl. Acad. Sci. U.S.A.* 109 E1848–E1857. 22645348 10.1073/pnas.1200250109 PMC3390875

[B157] Liang D. Hsiao B. S. Chu B. (2007). Functional electrospun nanofibrous scaffolds for biomedical applications. *Adv. Drug Deliv. Rev.* 59 1392–1412. 10.1016/j.addr.2007.04.021 17884240 PMC2693708

[B158] Liaw N. Y. Zimmermann W. H. (2016). Mechanical stimulation in the engineering of heart muscle. *Adv. Drug Deliv. Rev.* 96 156–160. 10.1016/j.addr.2015.09.001 26362920

[B159] Liu H. Wang Y. Cui K. Guo Y. Zhang X. Qin J. (2019). Advances in hydrogels in organoids and organs-on-a-chip. *Adv Mater*. 31:e1902042. 10.1002/adma.201902042 31282047

[B160] Louch W. E. Koivumäki J. T. Tavi P. (2015). Calcium signalling in developing cardiomyocytes: implications for model systems and disease. *J. Physiol.* 593 1047–1063. 10.1113/jphysiol.2014.274712 25641733 PMC4358669

[B161] Lux M. Andrée B. Horvath T. Nosko A. Manikowski D. Hilfiker-Kleiner D. (2016). In vitro maturation of large-scale cardiac patches based on a perfusable starter matrix by cyclic mechanical stimulation. *Acta Biomater.* 30 177–187. 10.1016/j.actbio.2015.11.006 26546973

[B162] MacIver D. H. Partridge J. B. Agger P. Stephenson R. S. Boukens B. J. D. Omann C. (2018a). The end of the unique myocardial band: part II. Clinical and functional considerations. *Eur. J. Cardio-thoracic Surg.* 53 120–128. 10.1093/ejcts/ezx335 29029119

[B163] MacIver D. H. Stephenson R. S. Jensen B. Agger P. Sánchez-Quintana D. Jarvis J. C. (2018b). The end of the unique myocardial band: part I. Anatomical considerations. *Eur. J. Cardio-Thoracic. Surg.* 53 112–119. 10.1093/ejcts/ezx290 28958005

[B164] Macqueen L. A. Sheehy S. P. Chantre C. O. Zimmerman J. F. Pasqualini F. S. Liu X. (2018). A tissue-engineered scale model of the heart ventricle. *Nat. Biomed. Eng.* 2 930–941. 10.1038/s41551-018-0271-5 31015723 PMC6774355

[B165] Maiullari F. Costantini M. Milan M. Pace V. Chirivì M. Maiullari S. (2018). A multi-cellular 3D bioprinting approach for vascularized heart tissue engineering based on HUVECs and iPSC-derived cardiomyocytes. *Sci. Rep.* 8 1–15. 30201959 10.1038/s41598-018-31848-x PMC6131510

[B166] Majkut S. Idema T. Swift J. Krieger C. Liu A. Discher D. E. (2013). Heart-specific stiffening in early embryos parallels matrix and myosin expression to optimize beating. *Curr. Biol.* 23 2434–2439. 10.1016/j.cub.2013.10.057 24268417 PMC4116639

[B167] Mannhardt I. Warncke C. Trieu H. K. Müller J. Eschenhagen T. (2019). Piezo-bending actuators for isometric or auxotonic contraction analysis of engineered heart tissue. *J. Tissue Eng. Regen Med.* 13 3–11. 10.1002/term.2755 30334614

[B168] Marvin M. J. Di Rocco G. Gardiner A. Bush S. M. Lassar A. B. (2001). Inhibition of Wnt activity induces heart formation from posterior mesoderm. *Genes Dev.* 15 316–327. 10.1101/gad.855501 11159912 PMC312622

[B169] Masoumi N. Annabi N. Assmann A. Larson B. L. Hjortnaes J. Alemdar N. (2014). Tri-layered elastomeric scaffolds for engineering heart valve leaflets. *Biomaterials* 35 7774–7785. 10.1016/j.biomaterials.2014.04.039 24947233 PMC4114056

[B170] Massai D. Cerino G. Gallo D. Pennella F. Deriu M. A. Rodriguez A. (2013). Bioreactors as engineering support to treat cardiac muscle and vascular disease. *J. Healthc. Eng.* 4 329–370. 10.1260/2040-2295.4.3.329 23965594

[B171] Mastikhina O. Moon B. U. Williams K. Hatkar R. Gustafson D. Mourad O. (2020). Human cardiac fibrosis-on-a-chip model recapitulates disease hallmarks and can serve as a platform for drug testing. *Biomaterials* 233:119741. 10.1016/j.biomaterials.2019.119741 31927251

[B172] Mawad D. Mansfield C. Lauto A. Perbellini F. Nelson G. W. Tonkin J. (2016). A conducting polymer with enhanced electronic stability applied in cardiac models. *Sci. Adv.* 2:e1601007. 10.1126/sciadv.1601007 28138526 PMC5262463

[B173] Mazzoli A. (2013). Selective laser sintering in biomedical engineering. *Med. Biol. Eng. Comput.* 51 245–256. 10.1007/s11517-012-1001-x 23250790

[B174] McKenna W. J. Maron B. J. Thiene G. (2017). Classification, epidemiology, and global burden of cardiomyopathies. *Circ. Res.* 121 722–730. 10.1161/circresaha.117.309711 28912179

[B175] Meilhac S. M. Buckingham M. E. (2018). The deployment of cell lineages that form the mammalian heart. *Nat. Rev. Cardiol.* 15 705–724. 10.1038/s41569-018-0086-9 30266935

[B176] Meilhac S. M. Lescroart F. Blanpain C. D. Buckingham M. E. (2014). Cardiac cell lineages that form the heart. *Cold Spring. Harb. Perspect. Med.* 4:a013888. 10.1101/cshperspect.a013888 25183852 PMC4143102

[B177] Melchels F. P. W. Feijen J. Grijpma D. W. (2010). A review on stereolithography and its applications in biomedical engineering. *Biomaterials* 31 6121–6130. 10.1016/j.biomaterials.2010.04.050 20478613

[B178] Menasché P. (2018). Cell therapy trials for heart regeneration – Lessons learned and future directions. *Nat. Rev. Cardiol.* 15 659–671. 10.1038/s41569-018-0013-0 29743563

[B179] Menasché P. Alfieri O. Janssens S. McKenna W. Reichenspurner H. Trinquart L. (2008). The myoblast autologous grafting in ischemic cardiomyopathy (MAGIC) trial: first randomized placebo-controlled study of myoblast transplantation. *Circulation* 117 1189–1200. 10.1161/circulationaha.107.734103 18285565

[B180] Menasché P. Vanneaux V. Hagège A. Bel A. Cholley B. Cacciapuoti I. (2015). Human embryonic stem cell-derived cardiac progenitors for severe heart failure treatment: first clinical case report. *Eur. Heart J.* 36 2011–2017. 10.1093/eurheartj/ehv189 25990469

[B181] Menasché P. Vanneaux V. Hagège A. Bel A. Cholley B. Parouchev A. (2018). Transplantation of human embryonic stem cell–derived cardiovascular progenitors for severe ischemic left ventricular dysfunction. *J. Am. Coll Cardiol.* 71 429–438. 10.1016/j.jacc.2017.11.047 29389360

[B182] Millard D. Dang Q. Shi H. Zhang X. Strock C. Kraushaar U. (2018). Cross-site reliability of human induced pluripotent stem cell-derived cardiomyocyte based safety assays using microelectrode arrays: results from a blinded cipa pilot study. *Toxicol. Sci.* 164 550–562. 10.1093/toxsci/kfy110 29718449 PMC6061700

[B183] Miller L. W. Guglin M. Rogers J. (2013). Cost of ventricular assist devices can we afford the progress? *Circulation* 127 743–748. 10.1161/circulationaha.112.139824 23401115

[B184] Miyagawa S. Domae K. Kainuma S. Matsuura R. Yoshioka D. Hata H. (2018). Long-term outcome of a dilated cardiomyopathy patient after mitral valve surgery combined with tissue-engineered myoblast sheets—report of a case. *Surg. Case Rep.* 4 1–5. 30547236 10.1186/s40792-018-0549-6 PMC6292833

[B185] Mjaatvedt C. H. Nakaoka T. Moreno-Rodriguez R. Norris R. A. Kern M. J. Eisenberg C. A. (2001). The outflow tract of the heart is recruited from a novel heart-forming field. *Dev. Biol.* 238 97–109. 10.1006/dbio.2001.0409 11783996

[B186] Mohamed T. M. A. Stone N. R. Berry E. C. Radzinsky E. Huang Y. Pratt K. (2017). Chemical enhancement of in vitro and in vivo direct cardiac reprogramming. *Circulation* 135 978–995. 10.1161/circulationaha.116.024692 27834668 PMC5340593

[B187] Mollova M. Bersell K. Walsh S. Savla J. Das L. T. Park S. Y. (2013). Cardiomyocyte proliferation contributes to heart growth in young humans. *Proc. Natl. Acad. Sci. U.S.A.* 110 1446–1451. 10.1073/pnas.1214608110 23302686 PMC3557060

[B188] Mommersteeg M. T. M. Domínguez J. N. Wiese C. Norden J. De Gier-De Vries C. Burch J. B. E. (2010). The sinus venosus progenitors separate and diversify from the first and second heart fields early in development. *Cardiovasc. Res.* 87 92–101. 10.1093/cvr/cvq033 20110338

[B189] Mooney D. J. Baldwin D. F. Suh N. P. Vacanti J. P. Langer R. (1996). Novel approach to fabricate porous sponges of poly(D,L-lactic-co-glycolic acid) without the use of organic solvents. *Biomaterials* 17 1417–1422. 10.1016/0142-9612(96)87284-x 8830969

[B190] Moretti A. Bellin M. Welling A. Jung C. B. Lam J. T. Bott-Flügel L. (2010). Patient-specific induced pluripotent stem-cell models for long-QT syndrome. *New. Engl. J. Med.* 363 1397–1409. 20660394 10.1056/NEJMoa0908679

[B191] Moroni L. De Wijn J. R. Van Blitterswijk C. A. (2006). 3D fiber-deposited scaffolds for tissue engineering: influence of pores geometry and architecture on dynamic mechanical properties. *Biomaterials* 27 974–985. 10.1016/j.biomaterials.2005.07.023 16055183

[B192] Mummery C. Ward-van Oostwaard D. Doevendans P. Spijker R. Van den Brink S. Hassink R. (2003). Differentiation of human embryonic stem cells to cardiomyocytes: role of coculture with visceral endoderm-like cells. *Circulation* 107 2733–2740. 10.1161/01.cir.0000068356.38592.68 12742992

[B193] Myers B. Dubick M. A. Keen C. L. Rucker R. B. (1985). Elastin synthesis during perinatal lung development in the rat. *Exp. Lung Res.* 8 227–241. 10.3109/01902148509087806 2864245

[B194] Nakamura M. Sadoshima J. (2018). Mechanisms of physiological and pathological cardiac hypertrophy. *Nat. Rev. Cardiol.* 15 387–407. 10.1038/s41569-018-0007-y 29674714

[B195] Nam Y. S. Park T. G. (1999). Porous biodegradable polymeric scaffolds prepared by thermally induced phase separation. *J. Biomed. Mater. Res.* 47 8–17. 10.1002/(sici)1097-4636(199910)47:1<8::aid-jbm2>3.0.co;2-l 10400875

[B196] Neejy J. R. (1974). Relationship between carbohydrate and lipid metabolism and the energy balance of heart muscle. *Annu. Rev. Physiol.* 36 413–459. 10.1146/annurev.ph.36.030174.002213 19400669

[B197] Nguyen-Truong M. Wang Z. (2018). Biomechanical properties and mechanobiology of cardiac ECM. *Adv. Exp. Med. Biol.* 1098 1–19. 10.1007/978-3-319-97421-7_1 30238363

[B198] Noor N. Shapira A. Edri R. Gal I. Wertheim L. Dvir T. (2019). 3D Printing of personalized thick and perfusable cardiac patches and hearts. *Adv. Sci.* 6:1900344. 10.1002/advs.201900344 31179230 PMC6548966

[B199] Nowbar A. N. Gitto M. Howard J. P. Francis D. P. Al-Lamee R. (2019). Mortality from ischemic heart disease: analysis of data from the world health organization and coronary artery disease risk factors from NCD risk factor collaboration. *Circ. Cardiovasc. Qual. Outcomes* 12 1–11. 10.1161/CIRCOUTCOMES.118.005375 PMC6613716 31163980

[B200] Nunes S. S. Miklas J. W. Liu J. Aschar-Sobbi R. Xiao Y. Zhang B. (2013). Biowire: a platform for maturation of human pluripotent stem cell-derived cardiomyocytes. *Nat. Methods* 10 781–787. 23793239 10.1038/nmeth.2524 PMC4071061

[B201] O’Brien F. J. Harley B. A. Yannas I. V. Gibson L. (2004). Influence of freezing rate on pore structure in freeze-dried collagen-GAG scaffolds. *Biomaterials* 25 1077–1086. 10.1016/s0142-9612(03)00630-6 14615173

[B202] Odde D. J. Renn M. J. (2000). Laser-guided direct writing of living cells. *Biotechnol. Bioeng.* 67 312–318. 10.1002/(sici)1097-0290(20000205)67:3<312::aid-bit7>3.0.co;2-f 10620261

[B203] Olvera D. Molina M. Hendy G. Monaghan M. (2020). Electroconductive melt electrowritten patches matching the mechanical anisotropy of human myocardium. *Adv. Funct. Mater.* 1909880.

[B204] Orive G. Taebnia N. Dolatshahi-Pirouz A. (2020). A new era for cyborg science is emerging: the promise of cyborganic beings. *Adv. Healthc. Mater.* 9:e1901023. 10.1002/adhm.201901023 31778037

[B205] Orive G. Tam S. K. Pedraz J. L. Hallé J. P. (2006). Biocompatibility of alginate-poly-l-lysine microcapsules for cell therapy. *Biomaterials* 27 3691–3700. 10.1016/j.biomaterials.2006.02.048 16574222

[B206] Orlova V. V. Van Den Hil F. E. Petrus-Reurer S. Drabsch Y. Ten Dijke P. Mummery C. L. (2014). Generation, expansion and functional analysis of endothelial cells and pericytes derived from human pluripotent stem cells. *Nat. Protoc.* 9 1514–1531. 10.1038/nprot.2014.102 24874816

[B207] Ott H. C. Matthiesen T. S. Goh S. K. Black L. D. Kren S. M. Netoff T. I. (2008). Perfusion-decellularized matrix: using nature’s platform to engineer a bioartificial heart. *Nat. Med.* 14 213–221. 10.1038/nm1684 18193059

[B208] Ouyang L. Highley C. B. Sun W. Burdick J. A. (2017). A generalizable strategy for the 3D bioprinting of hydrogels from nonviscous photo-crosslinkable inks. *Adv. Mater.* 29:1604983. 10.1002/adma.201604983 27982464

[B209] Pagano F. Picchio V. Chimenti I. Sordano A. De Falco E. Peruzzi M. (2019). On the road to regeneration: “tools” and “routes” towards efficient cardiac cell therapy for ischemic cardiomyopathy. *Curr. Cardiol. Rep.* 21:133. 10.1007/s11886-019-1226-5 31673821

[B210] Paige S. L. Osugi T. Afanasiev O. K. Pabon L. Reinecke H. Murry C. E. (2010). Endogenous wnt/β-Catenin signaling is required for cardiac differentiation in human embryonic stem cells. *PLoS ONE* 5:e11134. 10.1371/journal.pone.0011134 20559569 PMC2886114

[B211] Palpant N. J. Pabon L. Friedman C. E. Roberts M. Hadland B. Zaunbrecher R. J. (2017). Generating high-purity cardiac and endothelial derivatives from patterned mesoderm using human pluripotent stem cells. *Nat. Protoc.* 12 15–31. 10.1038/nprot.2016.153 27906170 PMC5576871

[B212] Parks W. C. Secrist H. Wu L. C. Mecham R. P. (1988). Developmental regulation of tropoelastin isoforms. *J. Biol. Chem.* 263 4416–4423. 3346253

[B213] Parmley W. W. Chuck L. Kivowitz C. Matloff J. M. Swan H. J. C. (1973). In vitro length-tension relations of human ventricular aneurysms: relation of stiffness to mechanical disadvantage. *Am. J. Cardiol.* 32 889–894. 10.1016/s0002-9149(73)80153-5 4271258

[B214] Parsa H. Ronaldson K. Vunjak-Novakovic G. (2016). Bioengineering methods for myocardial regeneration. *Adv. Drug Deliv. Rev.* 96 195–202. 10.1016/j.addr.2015.06.012 26150344 PMC4698189

[B215] Patsch C. Challet-Meylan L. Thoma E. C. Urich E. Heckel T. O’Sullivan J. F. (2015). Generation of vascular endothelial and smooth muscle cells from human pluripotent stem cells. *Nat. Cell Biol.* 17 994–1003. 26214132 10.1038/ncb3205 PMC4566857

[B216] Peña B. Laughter M. Jett S. Rowland T. J. Taylor M. R. G. Mestroni L. (2018). Injectable hydrogels for cardiac tissue engineering. *Macromol. Biosci.* 18 1–22. 10.1002/mabi.201800079 PMC6166441 29733514

[B217] Pérez-Pomares J. M. Phelps A. Sedmerova M. Carmona R. González-Iriarte M. Muoz-Chápuli R. (2002). Experimental studies on the spatiotemporal expression of WT1 and RALDH2 in the embryonic avian heart: a model for the regulation of myocardial and valvuloseptal development by epicardially derived cells (EPDCs). *Dev. Biol.* 247 307–326. 10.1006/dbio.2002.0706 12086469

[B218] Pinto A. R. Ilinykh A. Ivey M. J. Kuwabara J. T. D’antoni M. L. Debuque R. (2016). Revisiting cardiac cellular composition. *Circ. Res.* 118 400–409. 10.1161/circresaha.115.307778 26635390 PMC4744092

[B219] Pollock J. Baule V. J. Rich C. B. Ginsburg C. D. Curtiss S. W. Foster J. A. (1990). Chick tropoelastin isoforms. From the gene to the extracellular matrix. *J. Biol. Chem*. 265, 3697–3702. 2303474

[B220] Poveda F. Gil D. Martí E. Andaluz A. Ballester M. Carreras F. (2013). Helical structure of the cardiac ventricular anatomy assessed by diffusion tensor magnetic resonance imaging with multiresolution tractography. *Rev. Española Cardiol. (English Ed.)* 66 782–790. 10.1016/j.rec.2013.04.021 24773858

[B221] Prabhu P. K. (2019). Is presumed consent an ethically acceptable way of obtaining organs for transplant? *J. Intensive Care Soc.* 20 92–97. 10.1177/1751143718777171 31037100 PMC6475984

[B222] Prakash Y. S. Cody M. J. Housmans P. R. Hannon J. D. Sieck G. C. (1999). Comparison of cross-bridge cycling kinetics in neonatal vs. adult rat ventricular muscle. *J. Muscle Res. Cell Motil.* 20 717–723. 10672520 10.1023/a:1005585807179

[B223] Protze S. I. Liu J. Nussinovitch U. Ohana L. Backx P. H. Gepstein L. (2017). Sinoatrial node cardiomyocytes derived from human pluripotent cells function as a biological pacemaker. *Nat. Biotechnol.* 35 56–68. 10.1038/nbt.3745 27941801

[B224] Radisic M. Park H. Shing H. Consi T. Schoen F. J. Langer R. (2004a). Functional assembly of engineered myocardium by electrical stimulation of cardiac myocytes cultured on scaffolds. *Proc. Natl. Acad. Sci. U.S.A.* 101 18129–18134. 10.1073/pnas.0407817101 15604141 PMC539727

[B225] Radisic M. Yang L. Boublik J. Cohen R. J. Langer R. Freed L. E. (2004b). Medium perfusion enables engineering of compact and contractile cardiac tissue. *Am. J. Physiol. – Hear Circ. Physiol.* 286 507–516. 10.1152/ajpheart.00171.2003 14551059

[B226] Richards D. J. Tan Y. Coyle R. Li Y. Xu R. Yeung N. (2016). Nanowires and electrical stimulation synergistically improve functions of hiPSC cardiac spheroids. *Nano Lett.* 16 4670–4678. 10.1021/acs.nanolett.6b02093 27328393 PMC4994528

[B227] Ritz-Timme S. Laumeier I. Collins M. J. (2003). Aspartic acid racemization: evidence for marked longevity of elastin in human skin. *Br. J. Dermatol.* 149 951–959. 10.1111/j.1365-2133.2003.05618.x 14632798

[B228] Roberts D. E. Hersh L. T. Scher A. M. (1979). Influence of cardiac fiber orientation on wavefront voltage, conduction velocity, and tissue resistivity in the dog. *Circ Res.* 44 701–712. 10.1161/01.res.44.5.701 428066

[B229] Roberts W. C. Siegel R. J. McManus B. M. (1987). Idiopathic dilated cardiomyopathy: analysis of 152 necropsy patients. *Am. J. Cardiol.* 60 1340–1355. 10.1016/0002-9149(87)90618-7 3687784

[B230] Rock J. R. Onaitis M. W. Rawlins E. L. Lu Y. Clark C. P. Xue Y. (2009). Basal cells as stem cells of the mouse trachea and human airway epithelium. *Proc. Natl. Acad. Sci. U.S.A.* 106 12771–12775. 10.1073/pnas.0906850106 19625615 PMC2714281

[B231] Ronaldson-Bouchard K. Ma S. P. Yeager K. Chen T. Song L. J. Sirabella D. (2018). Advanced maturation of human cardiac tissue grown from pluripotent stem cells. *Nature* 1 239–243. 10.1038/s41586-018-0016-3 29618819 PMC5895513

[B232] Roshanbinfar K. Vogt L. Ruther F. Roether J. A. Boccaccini A. R. Engel F. B. (2020). Nanofibrous composite with tailorable electrical and mechanical properties for cardiac tissue engineering. *Adv. Funct. Mater.* 30:1908612.

[B233] Ruan J. L. Tulloch N. L. Razumova M. V. Saiget M. Muskheli V. Pabon L. (2016). Mechanical stress conditioning and electrical stimulation promote contractility and force maturation of induced pluripotent stem cell-derived human cardiac tissue. *Circulation* 134 1557–1567. 10.1161/circulationaha.114.014998 27737958 PMC5123912

[B234] Ruiz-Villalba A. Hoppler S. van den Hoff M. J. B. (2016). Wnt signaling in the heart fields: variations on a common theme. *Dev. Dyn.* 245 294–306. 10.1002/dvdy.24372 26638115

[B235] Sartiani L. Bettiol E. Stillitano F. Mugelli A. Cerbai E. Jaconi M. E. (2007). Developmental changes in cardiomyocytes differentiated from human embryonic stem cells: a molecular and electrophysiological approach. *Stem Cells* 25 1136–1144. 10.1634/stemcells.2006-0466 17255522

[B236] Sato S. Ashraf M. Millard R. W. Fujiwara H. Schwartz A. (1983). Connective tissue changes in early ischemia of porcine myocardium: an ultrastructural study. *J. Mol. Cell Cardiol.* 15 261–275. 10.1016/0022-2828(83)90281-x 6876183

[B237] Sauer H. Rahimi G. Hescheler J. Wartenberg M. (1999). Effects of electrical fields on cardiomyocyte differentiation of embryonic stem cells. *J. Cell Biochem.* 75 710–723. 10.1002/(sici)1097-4644(19991215)75:4<710::aid-jcb16>3.0.co;2-z 10572253

[B238] Schächinger V. Erbs S. Elsässer A. Haberbosch W. Hambrecht R. Hölschermann H. (2006). Intracoronary bone marrow-derived progenitor cells in acute myocardial infarction. *N. Engl. J. Med.* 355 1210–1221. 16990384 10.1056/NEJMoa060186

[B239] Scollan D. F. Holmes A. Winslow R. Forder J. (1998). Histological validation of myocardial microstructure obtained from diffusion tensor magnetic resonance imaging. *Am. J. Physiol. – Hear Circ Physiol.* 275 2308–2318. 10.1152/ajpheart.1998.275.6.H2308 9843833

[B240] Sensini A. Cristofolini L. (2018). Biofabrication of electrospun scaffolds for the regeneration of tendons and ligaments. *Materials (Basel)* 11 1–43. 10.3390/ma11101963 PMC6213815 30322082

[B241] Sephel G. C. Buckley A. Davidson J. M. (1987). Developmental initiation of elastin gene expression by human fetal skin fibroblasts. *J. Invest. Dermatol.* 88 732–735. 10.1111/1523-1747.ep12470403 3585057

[B242] Serena E. Figallo E. Tandon N. Cannizzaro C. Gerecht S. Elvassore N. (2009). Electrical stimulation of human embryonic stem cells: cardiac differentiation and the generation of reactive oxygen species. *Exp. Cell Res.* 315 3611–3619. 10.1016/j.yexcr.2009.08.015 19720058 PMC2787733

[B243] Serra M. Correia C. Malpique R. Brito C. Jensen J. Bjorquist P. (2011). Microencapsulation technology: a powerful tool for integrating expansion and cryopreservation of human embryonic stem cells. *PLoS One* 6:e23212. 10.1371/journal.pone.0023212 21850261 PMC3151290

[B244] Sessions A. O. Engler A. J. (2016). Mechanical regulation of cardiac aging in model systems. *Circ Res.* 118 1553–1562. 10.1161/circresaha.116.307472 27174949 PMC4868502

[B245] Shadrin I. Y. Allen B. W. Qian Y. Jackman C. P. Carlson A. L. Juhas M. E. (2017). Cardiopatch platform enables maturation and scale-up of human pluripotent stem cell-derived engineered heart tissues. *Nat. Commun.* 8:1825. 10.1038/s41467-017-01946-x PMC5705709 29184059

[B246] Sharma A. Sances S. Workman M. J. Svendsen C. N. (2020). Multi-lineage human iPSC-derived platforms for disease modeling and drug discovery. *Cell Stem Cell* 26 309–329. 10.1016/j.stem.2020.02.011 32142662 PMC7159985

[B247] Shin S. R. Jung S. M. Zalabany M. Kim K. Zorlutuna P. Kim S. B. (2013). Carbon-nanotube-embedded hydrogel sheets for engineering cardiac constructs and bioactuators. *ACS Nano* 7 2369–2380. 10.1021/nn305559j 23363247 PMC3609875

[B248] Sireesha M. Jagadeesh Babu V. Ramakrishna S. (2015). Biocompatible and biodegradable elastomer/fibrinogen composite electrospun scaffolds for cardiac tissue regeneration. *RSC Adv.* 5 103308–103314. 10.1039/c5ra20322h

[B249] Sizarov A. Lamers W. H. Mohun T. J. Brown N. A. Anderson R. H. Moorman A. F. M. (2012). Three-dimensional and molecular analysis of the arterial pole of the developing human heart. *J. Anat.* 220 336–349. 10.1111/j.1469-7580.2012.01474.x 22296102 PMC3375770

[B250] Solaro R. J. (2007). Mechanisms of the frank-starling law of the heart: the beat goes on. *Biophys. J.* 93 4095–4096. 10.1529/biophysj.107.117200 17766360 PMC2098723

[B251] Sommer G. Haspinger D. C. Andrä M. Sacherer M. Viertler C. Regitnig P. (2015a). Quantification of shear deformations and corresponding stresses in the biaxially tested human myocardium. *Ann. Biomed. Eng.* 43 2334–2348. 10.1007/s10439-015-1281-z 25707595

[B252] Sommer G. Schriefl A. J. Andrä M. Sacherer M. Viertler C. Wolinski H. (2015b). Biomechanical properties and microstructure of human ventricular myocardium. *Acta Biomater.* 24 172–192. 10.1016/j.actbio.2015.06.031 26141152

[B253] Spach M. S. Heidlage J. F. (1995). The stochastic nature of cardiac propagation at a microscopic level. Electrical description of myocardial architecture and its application to conduction. *Circ Res.* 76 366–380. 10.1161/01.res.76.3.366 7859383

[B254] Stehlik J. Edwards L. B. Kucheryavaya A. Y. Benden C. Christie J. D. Dobbels F. (2011). The registry of the international society for heart and lung transplantation: twenty-eighth adult heart transplant report-2011. *J. Hear Lung Transplant.* 30 1078–1094. 10.1016/j.healun.2011.08.003 21962016

[B255] Stöhr A. Friedrich F. W. Flenner F. Geertz B. Eder A. Schaaf S. (2013). Contractile abnormalities and altered drug response in engineered heart tissue from Mybpc3-targeted knock-in mice. *J. Mol. Cell Cardiol.* 63 189–198. 10.1016/j.yjmcc.2013.07.011 23896226

[B256] Stoppel W. L. Kaplan D. L. Black L. D. (2016). Electrical and mechanical stimulation of cardiac cells and tissue constructs. *Adv. Drug Deliv. Rev.* 96 135–155. 10.1016/j.addr.2015.07.009 26232525 PMC4698182

[B257] Sussman M. A. McCulloch A. Borg T. K. (2002). Dance band on the Titanic: biomechanical signaling in cardiac hypertrophy. *Circ. Res.* 91 888–898. 10.1161/01.res.0000041680.43270.f8 12433833

[B258] Taccardi B. Punske B. B. Macchi E. MacLeod R. S. Ershler P. R. (2008). Epicardial and intramural excitation during ventricular pacing: effect of myocardial structure. *Am. J. Physiol. – Hear Circ Physiol.* 294 1753–1766. 10.1152/ajpheart.01400.2007 PMC2745833 18263708

[B259] Takahashi K. Yamanaka S. (2006). Induction of pluripotent stem cells from mouse embryonic and adult fibroblast cultures by defined factors. *Cell* 126 663–676. 10.1016/j.cell.2006.07.024 16904174

[B260] Tamay D. G. Usal T. D. Alagoz A. S. Yucel D. Hasirci N. Hasirci V. (2019). 3D and 4D printing of polymers for tissue engineering applications. *Front. Bioeng. Biotechnol.* 7:164. 10.3389/fbioe.2019.00164 31338366 PMC6629835

[B261] Tan S. H. Ye L. (2018). Maturation of pluripotent stemcell-derived cardiomyocytes: a critical step for drug development and cell therapy. *J. Cardiovasc. Transl. Res.* 11 375–392. 10.1007/s12265-018-9801-5 29557052

[B262] Tandon N. Cannizzaro C. Chao P. P.-H. G. Maidhof R. Marsano A. Au H. T. H. (2009). Electrical stimulation systems for cardiac tissue engineering. *Nat. Protoc.* 4 155–173. 19180087 10.1038/nprot.2008.183 PMC2775058

[B263] Tandon N. Marsano A. Cannizzaro C. Voldman J. Vunjak-Novakovic G. (2008). Design of electrical stimulation bioreactors for cardiac tissue engineering. *Conf. Proc. IEEE Eng. Med. Biol. Soc.* 2008 3594–3597. 19163486 10.1109/IEMBS.2008.4649983 PMC2771167

[B264] Thomson J. A. Itskovitz-eldor J. Shapiro S. S. Michelle A. Swiergiel J. J. Marshall V. S. (1998). Embryonic stem cell lines derived from human blastocysts. *Science (80-)* 282 1145–1147. 10.1126/science.282.5391.1145 9804556

[B265] Thomson R. C. Yaszemski M. J. Powers J. M. Mikos A. G. (1995). Fabrication of biodegradable polymer scaffolds to engineer trabecular bone. *J. Biomater. Sci. Polym. Ed.* 7 23–38. 10.1163/156856295x00805 7662615

[B266] Tiburcy M. Hudson J. E. Balfanz P. Schlick S. Meyer T. Liao M. L. C. (2017). Defined engineered human myocardium with advanced maturation for applications in heart failure modeling and repair. *Circulation* 9 1832–1847. 10.1161/circulationaha.116.024145 28167635 PMC5501412

[B267] Tohyama S. Hattori F. Sano M. Hishiki T. Nagahata Y. Matsuura T. (2013). Distinct metabolic flow enables large-scale purification of mouse and human pluripotent stem cell-derived cardiomyocytes. *Cell Stem Cell* 12 127–137. 10.1016/j.stem.2012.09.013 23168164

[B268] Townsend N. Wilson L. Bhatnagar P. Wickramasinghe K. Rayner M. Nichols M. (2016). Cardiovascular disease in Europe: epidemiological update 2016. *Eur. Heart J.* 37 3232–3245. 10.1093/eurheartj/ehw334 27523477 PMC13480964

[B269] Tran T. H. Wang X. Browne C. Zhang Y. Schinke M. Izumo S. (2009). Wnt3a-induced mesoderm formation and cardiomyogenesis in human embryonic stem cells. *Stem Cells* 27 1869–1878. 10.1002/stem.95 19544447

[B270] Ueno S. Weidinger G. Osugi T. Kohn A. D. Golob J. L. Pabon L. (2007). Biphasic role for Wnt/β-catenin signaling in cardiac specification in zebrafish and embryonic stem cells. *Proc. Natl. Acad. Sci. U.S.A.* 104 9685–9690. 10.1073/pnas.0702859104 17522258 PMC1876428

[B271] Ulmer B. M. Stoehr A. Schulze M. L. Patel S. Gucek M. Mannhardt I. (2018). Contractile work contributes to maturation of energy metabolism in hiPSC-derived cardiomyocytes. *Stem Cell Rep.* 10 834–847. 10.1016/j.stemcr.2018.01.039 29503093 PMC5919410

[B272] Uosaki H. Fukushima H. Takeuchi A. Matsuoka S. Nakatsuji N. Yamanaka S. (2011). Efficient and scalable purification of cardiomyocytes from human embryonic and induced pluripotent stem cells by VCAM1 surface expression. *PLoS ONE* 6:e23657. 10.1371/journal.pone.0023657 21876760 PMC3158088

[B273] Vaithilingam J. Sanjuan-Alberte P. Campora S. Rance G. A. Jiang L. Thorpe J. (2019). Multifunctional bioinstructive 3D architectures to modulate cellular behavior. *Adv. Funct. Mater.* 29:1902016.

[B274] Valiente-Alandi I. Potter S. J. Salvador A. M. Schafer A. E. Schips T. Carrillo-Salinas F. (2018). Inhibiting fibronectin attenuates fibrosis and improves cardiac function in a model of heart failure. *Circulation* 138 1236–1252. 10.1161/circulationaha.118.034609 29653926 PMC6186194

[B275] Vallier L. Mendjan S. Brown S. Ching Z. Teo A. Smithers L. E. (2009). Activin/Nodal signalling maintains pluripotency by controlling Nanog expression. *Development* 136 1339–1349. 10.1242/dev.033951 19279133 PMC2687465

[B276] Valls-Margarit M. Iglesias-García O. Di Guglielmo C. Sarlabous L. Tadevosyan K. Paoli R. (2019). Engineered macroscale cardiac constructs elicit human myocardial tissue-like functionality. *Stem Cell Rep.* 9 207–220. 10.1016/j.stemcr.2019.05.024 31231023 PMC6626888

[B277] Vincent S. D. Buckingham M. E. (2010). How to make a heart. The origin and regulation of cardiac progenitor cells. *Curr. Top. Dev. Biol.* 90 1–41. 20691846 10.1016/S0070-2153(10)90001-X

[B278] Vozzi F. Logrand F. Cabiati M. Cicione C. Boffito M. Carmagnola I. (2018). Biomimetic engineering of the cardiac tissue through processing, functionalization, and biological characterization of polyester urethanes. *Biomed. Mater.* 13:055006. 10.1088/1748-605x/aaca5b 29869614

[B279] Vreeker A. Van Stuijvenberg L. Hund T. J. Mohler P. J. Nikkels P. G. J. Van Veen T. A. B. (2014). Assembly of the cardiac intercalated disk during preand postnatal development of the human heart. *PLoS ONE* 9:e94722. 10.1371/journal.pone.0094722 24733085 PMC3986238

[B280] Vunjak-Novakovic G. Eschenhagen T. Mummery C. (2014). Myocardial tissue engineering: in vitro models. *Cold Spring Harb. Perspect. Med.* 4 1–16. 10.1101/cshperspect.a014076 PMC3935388 24591534

[B281] Walker B. W. Lara R. P. Yu C. H. Sani E. S. Kimball W. Joyce S. (2019). Engineering a naturally-derived adhesive and conductive cardiopatch. *Biomaterials* 1 89–101. 10.1016/j.biomaterials.2019.03.015 30965152 PMC6470010

[B282] Walker I. F. Garbe F. Wright J. Newell I. Athiraman N. Khan N. (2018). *The Economic Costs of Cardiovascular Disease, Diabetes Mellitus, and Associated Complications in South Asia: A Systematic Review.* Available online at: http://eprints.whiterose.ac.uk/118785/ (accessed June 16, 2020). 10.1016/j.vhri.2017.05.003 29474174

[B283] Wang Y. Yao F. Wang L. Li Z. Ren Z. Li D. (2020). Single-cell analysis of murine fibroblasts identifies neonatal to adult switching that regulates cardiomyocyte maturation. *Nat. Commun.* 11:2585. 10.1038/s41467-020-16204-w PMC7244751 32444791

[B284] Wang Z. Gagliardi M. Mohamadi R. M. Ahmed S. U. Labib M. Zhang L. (2020). Ultrasensitive and rapid quantification of rare tumorigenic stem cells in hPSC-derived cardiomyocyte populations. *Sci. Adv.* 6 1–12. 10.1126/sciadv.aay7629 PMC7227422 32440533

[B285] Watson S. A. Duff J. Bardi I. Zabielska M. Atanur S. S. Jabbour R. J. (2019). Biomimetic electromechanical stimulation to maintain adult myocardial slices in vitro. *Nat. Commun.* 10:2168. 10.1038/s41467-019-10175-3 PMC6520377 31092830

[B286] Weber K. T. (1989). Cardiac interstitium in health and disease: the fibrillar collagen network. *J. Am. Coll. Cardiol.* 13 1637–1652. 10.1016/0735-1097(89)90360-4 2656824

[B287] Weber K. T. Pick R. Janicki J. S. Gadodia G. Lakier J. B. (1988). Inadequate collagen tethers in dilated cardiopathy. *Am. Heart J.* 116 1641–1646. 10.1016/0002-8703(88)90763-6 3195449

[B288] Weeke-Klimp A. Bax N. A. M. Bellu A. R. Winter E. M. Vrolijk J. Plantinga J. (2010). Epicardium-derived cells enhance proliferation, cellular maturation and alignment of cardiomyocytes. *J. Mol. Cell Cardiol.* 49 606–616. 10.1016/j.yjmcc.2010.07.007 20655924

[B289] Weinberger F. Breckwoldt K. Pecha S. Kelly A. Geertz B. Starbatty J. (2016). Cardiac repair in Guinea pigs with human engineered heart tissue from induced pluripotent stem cells. *Sci. Transl. Med.* 8:363ra148. 10.1126/scitranslmed.aaf8781 27807283

[B290] White M. P. Rufaihah A. J. Liu L. Ghebremariam Y. T. Ivey K. N. Cooke J. P. (2013). Limited gene expression variation in human embryonic stem cell and induced pluripotent stem cell-derived endothelial cells. *Stem Cells* 31 92–103. 10.1002/stem.1267 23079999 PMC3528812

[B291] Wickline S. A. Verdonk Wong A. K. Shepard R. K. Miller J. G. (1992). Structural remodeling of human myocardial tissue after infarction, quantification with ultrasonic backscatter. *Circulation* 85 259–268. 10.1161/01.cir.85.1.259 1728457

[B292] Witty A. D. Mihic A. Tam R. Y. Fisher S. A. Mikryukov A. Shoichet M. S. (2014). Generation of the epicardial lineage from human pluripotent stem cells. *Nat. Biotechnol.* 32 1026–1035. 25240927 10.1038/nbt.3002 PMC4192149

[B293] Wollert K. C. Meyer G. P. Lotz J. Ringes-Lichtenberg S. Lippolt P. Breidenbach C. (2004). Randomised controlled trial of intracoroanry autologous bone marrow cell transfer after myocardial infarction. *Lancet* 2004 141–148. 10.1016/s0140-6736(04)16626-9 15246726

[B294] Woodall M. C. Woodall B. P. Gao E. Yuan A. Koch W. J. (2016). Cardiac fibroblast GRK2 deletion enhances contractility and remodeling following ischemia/reperfusion injury. *Circ. Res.* 119 1116–1127. 10.1161/circresaha.116.309538 27601479 PMC5085864

[B295] Woodruff M. A. Hutmacher D. W. (2010). The return of a forgotten polymer – Polycaprolactone in the 21st century. *Prog. Polym. Sci.* 35 1217–1256. 10.1016/j.progpolymsci.2010.04.002

[B296] World Health Organization [WHO] (2018). *Global Health Estimates 2016: Deaths by Cause, Age, Sex, by Country and by Region, 2000–2016.* Genoa: World Health Organization [WHO].

[B297] Wunner F. M. Wille M. L. Noonan T. G. Bas O. Dalton P. D. De-Juan-Pardo E. M. (2018). Melt electrospinning writing of highly ordered large volume scaffold architectures. *Adv. Mater.* 30 1–6. 10.1002/adma.201706570 29633443

[B298] Xu C. Dai G. Hong Y. (2019). Recent advances in high-strength and elastic hydrogels for 3D printing in biomedical applications. *Acta Biomater.* 95 50–59. 10.1016/j.actbio.2019.05.032 31125728 PMC6710142

[B299] Yamauchi K. Sumi T. Minami I. Otsuji T. G. Kawase E. Nakatsuji N. (2010). Cardiomyocytes develop from anterior primitive streak cells induced by β-catenin activation and the blockage of BMP signaling in hESCs. *Genes Cells* 15 1216–1227. 10.1111/j.1365-2443.2010.01455.x 21050342

[B300] Yang L. Soonpaa M. H. Adler Roepke T. K. Kattman S. J. Kennedy M. (2008). Human cardiovascular progenitor cells develop from a KDR+ embryonic-stem-cell-derived population. *Nature* 453 524–528. 10.1038/nature06894 18432194

[B301] Yang X. Pabon L. Murry C. E. (2014). Engineering adolescence: maturation of human pluripotent stem cell-derived cardiomyocytes. *Circ. Res* 114 511–523. 10.1161/circresaha.114.300558 24481842 PMC3955370

[B302] Yap L. Wang J. W. Moreno-Moral A. Chong L. Y. Sun Y. Harmston N. (2019). In vivo generation of post-infarct human cardiac muscle by laminin-promoted cardiovascular progenitors. *Cell Rep.* 26 3231.e9–3245.e9. 30893597 10.1016/j.celrep.2019.02.083

[B303] Young J. L. Engler A. J. (2011). Hydrogels with time-dependent material properties enhance cardiomyocyte differentiation in vitro. *Biomaterials* 32 1002–1009. 10.1016/j.biomaterials.2010.10.020 21071078 PMC3000555

[B304] Yu J. Vodyanik M. A. Smuga-Otto K. Antosiewicz-Bourget J. Frane J. L. Tian S. (2007). Induced pluripotent stem cell lines derived from human somatic cells. *Science (80-)* 318 1917–1920. 10.1126/science.1151526 18029452

[B305] Yu P. Pan G. Yu J. Thomson J. A. (2011). FGF2 sustains NANOG and switches the outcome of BMP4-induced human embryonic stem cell differentiation. *Cell Stem Cell* 8 326–334. 10.1016/j.stem.2011.01.001 21362572 PMC3052735

[B306] Yuasa S. Itabashi Y. Koshimizu U. Tanaka T. Sugimura K. Kinoshita M. (2005). Transient inhibition of BMP signaling by Noggin induces cardiomyocyte differentiation of mouse embryonic stem cells. *Nat. Biotechnol.* 23 607–611. 10.1038/nbt1093 15867910

[B307] Yue K. Trujillo-de Santiago G. Alvarez M. M. Tamayol A. Annabi N. Khademhosseini A. (2015). Synthesis, properties, and biomedical applications of gelatin methacryloyl (GelMA) hydrogels. *Biomaterials* 73 254–271. 26414409 10.1016/j.biomaterials.2015.08.045 PMC4610009

[B308] Zhang D. Shadrin I. Y. Lam J. Xian H. Q. Snodgrass H. R. Bursac N. (2013). Tissue-engineered cardiac patch for advanced functional maturation of human ESC-derived cardiomyocytes. *Biomaterials* 34 5813–5820. 10.1016/j.biomaterials.2013.04.026 23642535 PMC3660435

[B309] Zhang H. Tian L. Shen M. Tu C. Wu H. Gu M. (2019). Generation of quiescent cardiac fibroblasts from human induced pluripotent stem cells for in vitro modeling of cardiac fibrosis. *Circ. Res.* 125 552–566. 10.1161/circresaha.119.315491 31288631 PMC6768436

[B310] Zhang J. Tao R. Campbell K. F. Carvalho J. L. Ruiz E. C. Kim G. C. (2019). Functional cardiac fibroblasts derived from human pluripotent stem cells via second heart field progenitors. *Nat. Commun.* 10:2238. 10.1038/s41467-019-09831-5 PMC6527555 31110246

[B311] Zhang J. Wilson G. F. Soerens A. G. Koonce C. H. Yu J. Palecek S. P. (2009). Functional cardiomyocytes derived from human induced pluripotent stem cells. *Circ. Res.* 104 30–41. 10.1161/CIRCRESAHA.108.192237 PMC2741334 19213953

[B312] Zhang J. Zhu W. Radisic M. Vunjak-Novakovic G. (2018). Can we engineer a human cardiac patch for therapy? *Circ. Res.* 123 244–265. 10.1161/circresaha.118.311213 29976691 PMC7250155

[B313] Zhang L. G. Fisher J. Leong K. (2015). *3D Bioprinting and Nanotechnology in Tissue Engineering and Regenerative Medicine*, 1st Edn Cambridge: Academic Press, 60–68.

[B314] Zhang P. Li J. Tan Z. Wang C. Liu T. Chen L. (2008). Short-term BMP-4 treatment initiates mesoderm induction in human embryonic stem cells. *Blood* 111 1933–1941. 10.1182/blood-2007-02-074120 18042803

[B315] Zhang Y. Cao N. Huang Y. Spencer C. I. Fu J. D. Yu C. (2016). Expandable cardiovascular progenitor cells reprogrammed from fibroblasts. *Cell Stem Cell* 18 368–381. 10.1016/j.stem.2016.02.001 26942852 PMC5826660

[B316] Zhang Y. S. Arneri A. Bersini S. Shin S. R. Zhu K. Goli-Malekabadi Z. (2016). Bioprinting 3D microfibrous scaffolds for engineering endothelialized myocardium and heart-on-a-chip. *Biomaterials* 110 45–59. 10.1016/j.biomaterials.2016.09.003 27710832 PMC5198581

[B317] Zhao Y. Rafatian N. Feric N. T. Cox B. J. Aschar-Sobbi R. Wang E. Y. (2019). A platform for generation of chamber-specific cardiac tissues and disease modeling. *Cell* 176 913.e18–927.e18. 30686581 10.1016/j.cell.2018.11.042 PMC6456036

[B318] Zhou P. Pu W. T. (2016). Recounting cardiac cellular composition. *Circ. Res.* 118 368–370. 10.1161/circresaha.116.308139 26846633 PMC4755297

[B319] Zhu K. Shin S. R. van Kempen T. Li Y. C. Ponraj V. Nasajpour A. (2017). Gold nanocomposite bioink for printing 3D cardiac constructs. *Adv. Funct. Mater.* 27:1605352. 10.1002/adfm.201605352 30319321 PMC6181228

[B320] Zimmermann W. H. Fink C. Kralisch D. Remmers U. Weil J. Eschenhagen T. (2000). Three-dimensional engineered heart tissue from neonatal rat cardiac myocytes. *Biotechnol. Bioeng.* 68 106–114. 10.1002/(sici)1097-0290(20000405)68:1<106::aid-bit13>3.0.co;2-3 10699878

[B321] Zimmermann W. H. Melnychenko I. Wasmeier G. Didié M. Naito H. Nixdorff U. (2006). Engineered heart tissue grafts improve systolic and diastolic function in infarcted rat hearts. *Nat. Med.* 12 452–458. 10.1038/nm1394 16582915

[B322] Zimmermann W. H. Schneiderbanger K. Schubert P. Didié M. Münzel F. Heubach J. F. (2002). Tissue engineering of a differentiated cardiac muscle construct. *Circ. Res.* 90 223–230. 10.1161/hh0202.103644 11834716

[B323] Zócalo Y. Guevara E. Bia D. Giacche E. Pessana F. Peidro R. (2008). A reduction in the magnitude and velocity of left ventricular torsion may be associated with increased left ventricular efficiency: evaluation by speckle-tracking echocardiography. *Rev. española Cardiol.* 61 705–713. 18590643

